# AIEgen‐Based Proactive Early Warning System and Precise Treatment Strategy for Monkeypox Prevention and Control

**DOI:** 10.1002/advs.202515865

**Published:** 2025-12-05

**Authors:** Wei Wang, Zining Liu, Mengjun Li, Judun Zheng, Ruilin Zhang, Ke Liu, Xiaoxue Li, Shaoying Wen, Jingze Yu, Meijia Pan, Fengbo Ma, Kun Zhou, Zheng Zhao, Chenguang Shen, Yuhui Liao

**Affiliations:** ^1^ Institute for Engineering Medicine Kunming Medical University Kunming Yunnan 650500 P. R. China; ^2^ School of inspection Ningxia Medical University Yinchuan Ningxia 750004 P. R. China; ^3^ BSL‐3 Laboratory (Guangdong) Guangdong Provincial Key Laboratory of Tropical Disease Research School of Public Health Department of Laboratory Medicine Zhujiang Hospital，Key Laboratory of Infectious Diseases Research in South China, Southern Medical University, Ministry of Education Southern Medical University Guangzhou Guangdong 510515 P. R. China; ^4^ Guangdong Basic Research Center of Excellence for Aggregate Science Shenzhen Institute of Aggregate Science and Technology School of Science and Engineering The Chinese University of Hong Kong Shenzhen (CUHK‐Shenzhen) Guangdong 518172 P. R. China; ^5^ Key Laboratory of Infectious Diseases Research in South China (Southern Medical University) Ministry of Education Guangzhou Guangdong 510515 P. R. China

**Keywords:** AIEgen, control, monkeypox, photodynamic therapy, prevention, warning system

## Abstract

The recent monkeypox outbreak is garnering attention, but early warning and treatment mechanisms are lacking. Owing to its rapid spread and high death rate, monkeypox must be prevented and controlled immediately. An advanced early warning system for monkeypox prevention and control is provided through aggregation‐induced emission luminogen (AIEgen)‐active monitoring and precision photodynamic therapy (PDT). This system are developed by combining a sprayable AIE@LD with a commercial liquid dressing (LD) and a pyridine‐substituted benzothiadiazole derivative possessing AIEgen properties. In vivo tests revealed that 1 min of low‐power white light PDT at 5 mW cm^−^^2^ promoted viral eradication and lesion healing within one week. The system comprises dual optical channels that incorporate smartphone image recognition software and a cloud platform, combining image analysis, data transmission, infection monitoring, and PDT to provide a comprehensive solution for detecting, monitoring, treating, and interrupting the transmission of monkeypox. This system can considerably reduce the medical burden of monkeypox epidemics and provide innovative ideas and technological assistance for intelligent prevention and management.

## Graphical abstract

An intelligent early warning and treatment system for monkeypox, utilizing a sprayable AIEgen‐ liquid dressing (AIE@LD) that responds to lesion pH. Integrated with a smartphone app and cloud platform, it enables real‐time infection detection via fluorescence imaging and triggers on‐demand photodynamic therapy under white light, effectively eradicating the virus, accelerating healing, and blocking transmission in a user‐friendly, scalable manner.

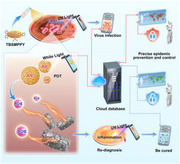

## 1 Introduction

In 2022, a global monkeypox (MPOX) outbreak occurred, rapidly spreading to more than 100 countries, making the situation extremely critical.^[^ ^1^, ^2^ ^]^ In August 2024, the epidemic re‐emerged in many countries, with the development of the highly virulent B.1.2 lineage leading to an increase in the fatality rate to 10%. The World Health Organization (WHO) reclassified the MPOX outbreak as a “Public Health Emergency of International Concern” (PHEIC), emphasizing its seriousness and the risk it poses to global health security.^[^ ^3^, ^4^ ^]^ This virus has a high capacity for sustained transmission and a high mutation rate, which could cause the outbreak to escalate uncontrollably and re‐emerge at any time.^[^ ^5^, ^6^, ^7^ ^]^ Consequently, it is imperative to implement an intelligent, proactive monitoring and early warning system that enables the effective identification, continuous monitoring, accurate treatment, and control of transmission, thereby mitigating the progression of the epidemic and preventing future outbreaks.

In response to the complex circumstances of the MPOX epidemic, countries primarily rely on conventional strategies, such as quarantine at entry points, surveillance in medical institutions, and health education for targeted groups, to establish a prevention and control framework.^[^ ^8^, ^9^ ^]^ However, these methods have several limitations, including failure to detect asymptomatic carriers, delayed monitoring, and inadequate coverage, which complicates the ability to achieve effective prevention and control.^[^ ^10^ ^]^ Prompt identification and ongoing dynamic surveillance are essential for controlling the transmission of infectious diseases.^[^ ^11^ ^]^ Nevertheless, current diagnostic methods often have drawbacks, including being time‐consuming, expensive, and dependent on specialized equipment, which limits the ability of primary healthcare institutions to respond rapidly and hinders epidemic prevention and control.^[^ ^12^, ^13^, ^14^ ^]^ Portable diagnostic devices for MPOX detection have yet to be developed, leaving patients reliant on medical facilities for diagnosis and treatment, which places additional strain on healthcare systems. Furthermore, existing patient tracking systems are inadequate, hindered by fragmented data sources, inconsistent formats, cumbersome data reporting procedures, and a lack of standardized data integration and management platforms.^[^ ^15^ ^]^ These issues significantly increase the complexity of data exchange and analysis, thereby affecting monitoring efficiency and accuracy.^[^ ^7^, ^16^, ^17^ ^]^ Therefore, developing a preventive and control system that integrates effective detection, patient self‐monitoring, standardized data management, and precise treatment to facilitate early detection, diagnosis, and intervention for MPOX is essential.

Aggregation‐induced emission luminogens (AIEgens) exhibit enhanced fluorescence in their aggregated forms, characterized by prolonged stability and improved signal‐to‐noise ratios,^[^ ^18^, ^19^, ^20^ ^]^ making them ideal for imaging bacteria, viruses, and tumours.^[^ ^18^, ^21^, ^22^, ^23^ ^]^ AIE‐based fluorescence sensors offer noninvasive, rapid, and highly specific diagnostic capabilities;^[^ ^24^, ^25^, ^26^ ^]^ however, there is currently no research on their use for MPOX detection. It is noteworthy that monkeypox virus‐infected cells enter the cell through interactions between viral ligands and glycosaminoglycans (such as chondroitin sulfate or heparan sulfate) acting as cell surface receptors, where fusion and uncoating occur in a low pH‐dependent manner.^[^ ^27^ ^]^ The low pH (<6) in acidified endosomes promotes viral particle entry.^[^ ^27^ ^]^ These phenomena indicate that monkeypox virus infection relies to some extent on an acidic environment. Additionally, monkeypox virus infection typically progresses through stages such as papules, vesicles, pustules, ulcers, pseudopustules, and scabs,^[^ ^28^, ^29^ ^]^ which can result in a mildly acidic lesion microenvironment, particularly during the ulcer and scabbing stages. These characteristics can serve as important biomarkers for the early detection of monkeypox skin lesions.^[^ ^30^, ^31^, ^32^, ^33^, ^34^ ^]^ Therefore, developing AIEgens with diagnostic and monitoring functions based on pH fluctuations in monkeypox lesions holds considerable research significance and potential applications for early warning systems. To achieve rapid, accurate, and sustained disease diagnosis, integrating intelligent systems with computer vision and machine learning algorithms represents a viable strategy.^[^ ^35^, ^36^ ^]^ Intelligent solutions enable infection detection by allowing users to capture images of lesions via their smartphones, providing a noninvasive, cost‐effective method for disease screening.^[^ ^37^ ^]^ Intelligent detection has been applied to infectious diseases, including malaria and fungal infections;^[^ ^38^ ^]^ however, no intelligent diagnostic methods currently exist for MPOX. The system could also incorporate standardized health data, which can be uploaded to a cloud platform in real‐time, facilitating sharing and unified analysis, thereby improving monitoring efficiency. In addition to early identification and surveillance, appropriate treatment is crucial for MPOX.^[^ ^39^, ^40^ ^]^ Current treatments remain largely supportive. Our team previously developed TPE‐BT‐DCTBT for the treatment of MPOX.^[^ ^39^, ^41^ ^]^ However, this approach relies on intravenous injections and photothermal therapy (PTT), which is invasive and poses a risk of skin burns, thereby affecting patient compliance. In contrast, photodynamic therapy (PDT) offers several advantages, including non‐invasiveness, simplicity, greater spatiotemporal selectivity, reduced resistance, and fewer side effects,^[^ ^42^, ^43^, ^44^ ^]^ making it a more attractive therapeutic option for MPOX. Given the complexities of the MPOX epidemic and the urgent need for global public health security, implementing a sophisticated prevention and control early warning system that encompasses epidemic detection, dynamic monitoring, targeted treatment, and transmission interruption is essential to mitigate its transmission risk and public health impact.

**Figure 1:**
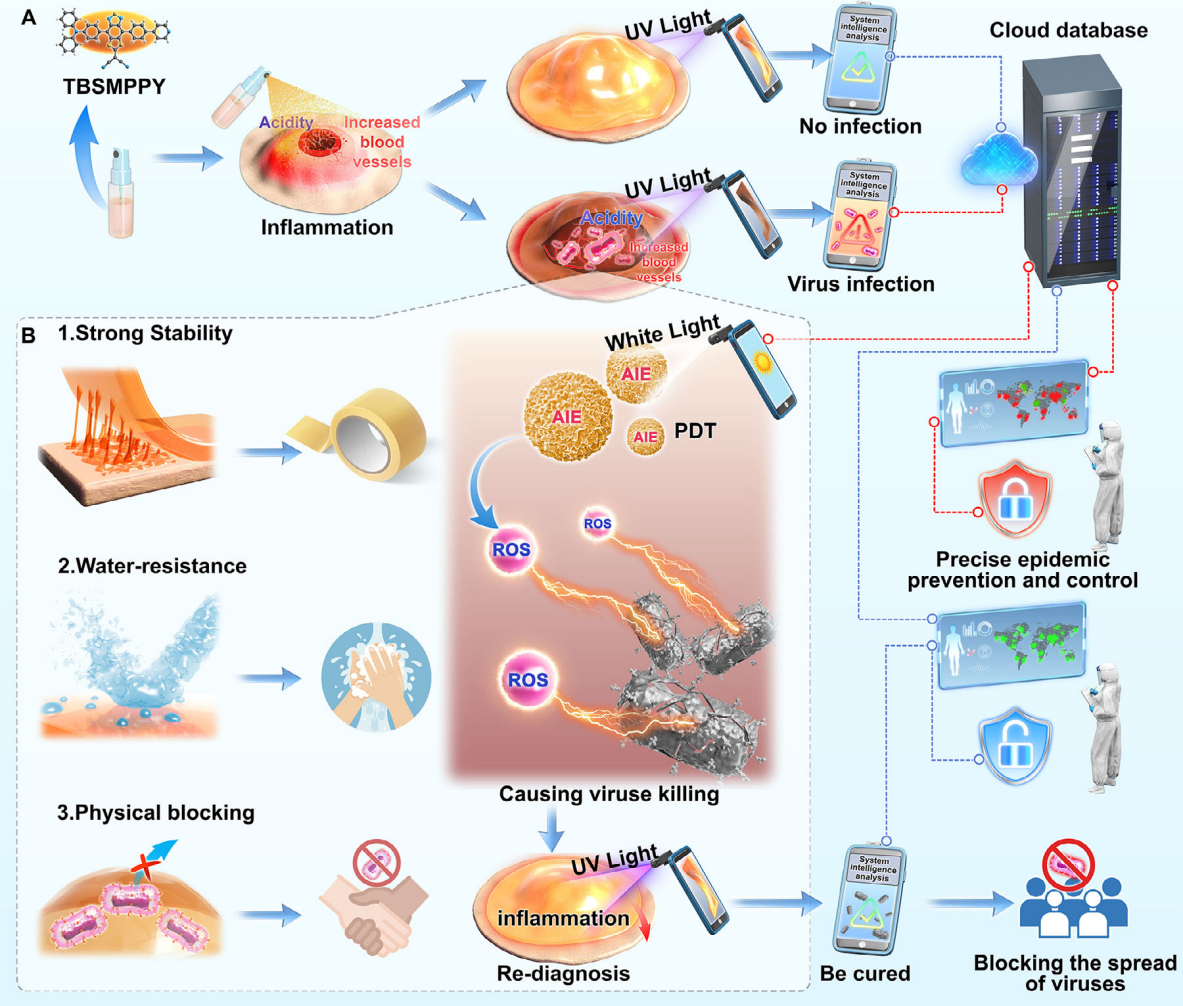
Schematic of the early intelligent warning system based on AIE@LD spray film and on‐demand photodynamic therapy. A) Active monitoring and a cloud platform for intelligent early warning systems. B) Photodynamic precision treatment and stability of AIE@LD.

In this study, we developed an intelligent early warning system based on a commercial liquid dressing (LD) as the substrate, integrated with a pyridine‐substituted benzothiadiazole derivative (TBSMPPy) that exhibits AIEgen properties (AIE@LD). This system combines active monitoring and PDT for precise treatment, along with a smartphone‐based image recognition system. The liquid dressing provides excellent adhesion, flexibility, and film‐forming characteristics, ensuring system stability while offering a physical barrier for MPOX lesions. Smartphones equipped with UV and white light sources act as excitation sources. After the AIE@LD spray is applied to the wound, photos are taken under UV light via the smartphone camera and sent to the cloud, creating a dynamic monitoring database. Once MPOX infection is detected, the system automatically initiates PDT treatment. If the wound emits a vivid orange light, indicating virus eradication, the system rescinds the warning and stops prevention and control measures, effectively controlling MPOX transmission. In vitro and in vivo studies demonstrated that this intelligent system can rapidly and accurately detect MPOX infections and efficiently eradicate the virus. Furthermore, the system offers excellent stability, biocompatibility, and ease of use. This system provides basic detection and monitoring methods, and through real‐time data uploads and cloud analysis, it offers novel support for the MPOX epidemic response. These measures can significantly reduce the burden on medical resources and establish an intelligent defence mechanism for public health safety (**Figure** **1**).

## 2 Results and Discussion

### 2.1 Characterization and pH Responsiveness of AIE@LD

**Figure 2:**
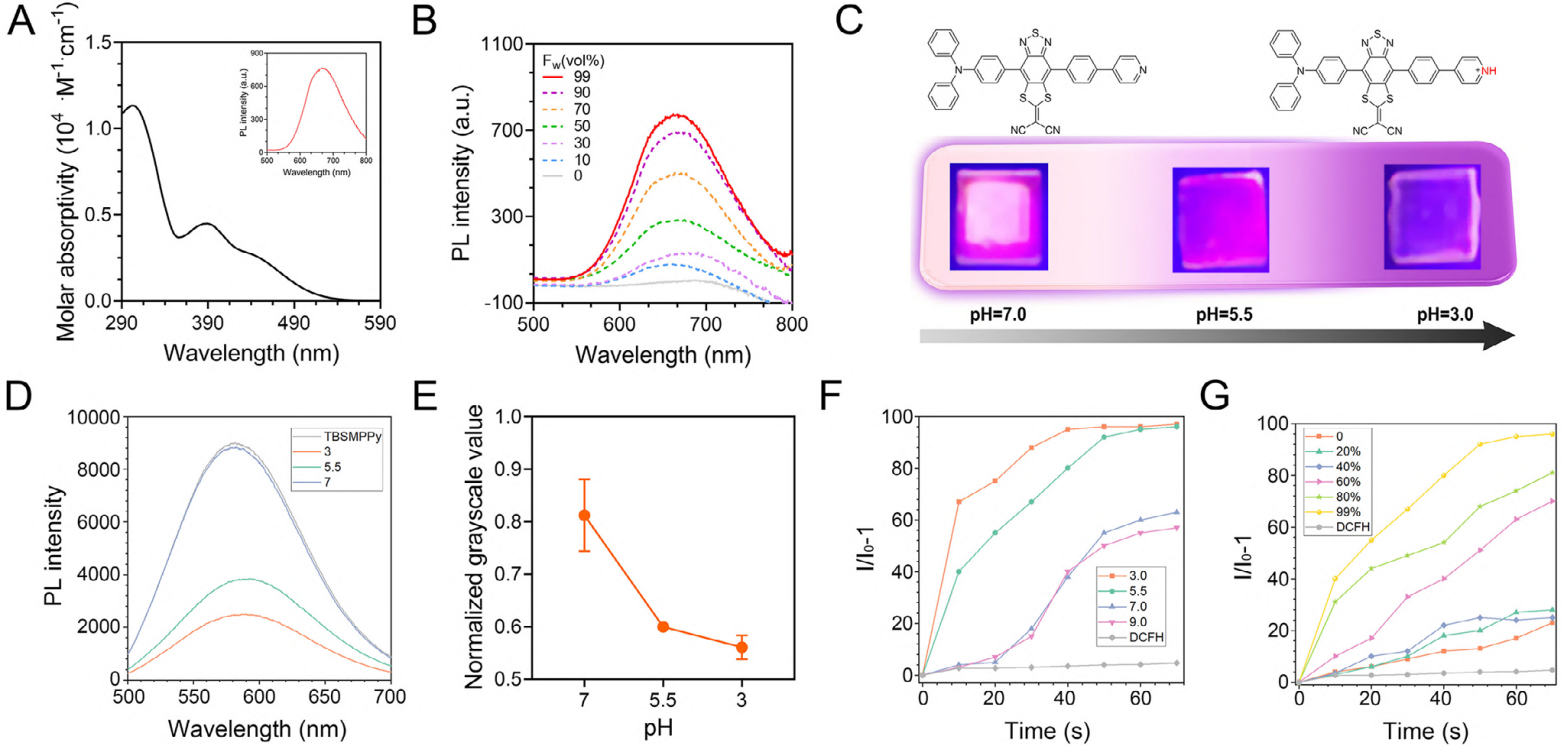
Characterization and pH responsiveness of AIE@LD. A) UV–vis absorption and photoluminescence (PL) spectra of AIE@LD. B) PL spectra of TBSMPPy in DMSO/water mixtures with different water (f_W_) ratios. C) Schematic diagram of the pH response mechanism of AIE@LD films and sample photos under UV light in different pH environments. D) Comparison of the PL spectra of AIE@LD under different pH conditions. E) Changes in the normalized grayscale values of AIE@LD films under different pH conditions. F) Changes in the relative PL intensity of 2′,7′‐dichlorodihydrofluorescein (DCFH) when irradiated with white light (5 mW cm^−^^2^) by AIE@LD under different pH conditions, where I_0_ and I represent the PL intensities of the indicator before and after irradiation, respectively. G) Changes in the relative PL intensity of DCFH under the action of AIE@LD in different fW DMSO/water mixtures. White‐light irradiation conditions: 5 mW cm^−^^2^. The AIE@LD stock solution concentration was 1 mm, and the final working concentration was 10 mm.

We previously designed and synthesized the organic photosensitizer TBSMPPy and effectively utilized it for treating fungus‐infected wounds.^[^ ^45^ ^]^ Under low‐power white‐light irradiation (5 mW cm^−^^2^), a single 1‐min photodynamic therapy (PDT) session achieved over 96% antifungal efficacy and significant wound healing within one week. Owing to the excellent acid responsiveness and fluorescence properties of TBSMPPy, we incorporated 1 mol of TBSMPPy into 1 mL of LD to prepare the spray film AIE@LD, which was then applied to MPOX lesions. TBSMPPy was synthesized according to the synthetic route shown in Scheme S1 (Supporting Information) and characterized by ^1^H, ^13^C nuclear magnetic resonance (NMR) and high‐resolution mass spectrometry (Figures S1–S3, Supporting Information). TBSMPpy maintained good UV–vis absorption and stable fluorescence characteristics after simulated incubation for 10 days (Figure S4, Supporting Information), which indicated that TBSMPy had good stability in a physiological environment. TBSMPPy consistently exhibited broad visible absorption in both LDs (**Figure** **2**A) and DMSO (Figure S5, Supporting Information). The photoluminescence (PL) spectrum of TBSMPPy shows an emission range from 530 to 800 nm, with a maximum at 678 nm, indicating orange fluorescence. Furthermore, as shown in Figure 2B, increasing the water fraction led to the formation of aggregates and a gradual increase in the PL intensity, which was significantly greater than that of TBSMPPy in the DMSO solution, confirming its aggregation‐induced emission (AIE) properties and suggesting substantial potential for film‐based applications. TBSMPPy also exhibited outstanding acid responsiveness and fluorescence characteristics. We conducted pH response studies on AIE@LD to investigate its fluorescence behavior under various acidic and basic conditions. The AIE@LD films were exposed to a range of pH environments and imaged under UV light, and their photoluminescence changes were recorded. The experimental results indicate that AIE@LD exhibits macroscopic fluorescence quenching in response to a decreasing pH (Figure 2C). At a pH of 7.0, the film emits bright orange fluorescence; however, its fluorescence significantly decreases as the pH decreases to 3.0. Upon exposure to a pH 5.5 buffer solution, the photoluminescence intensity of AIE@LD decreased rapidly (Figure 2D). Subsequent quantitative analysis revealed that a decrease in pH corresponds to a gradual decrease in the grayscale value of the film (Figure 2E). The PL change with significantly reduced AIE@LD activity is mainly attributed to protonation of the pyridine of TBSMPPY in low pH buffer solution, which enhances the electron‐withdrawing ability of the pyridine moiety. As shown in Figure S6 (Supporting Information), after protonation, the UV spectrum shows a distinct red shift, indicating a strong ICT effect. In addition, DFT calculations also show significant HOMO and LUMO separation, which suggests ICT effects enhance post‐protonation (Figure S7, Supporting Information). These results demonstrate the exceptional pH‐dependent fluorescence response of AIE@LD, highlighting its potential for detecting and monitoring MPOX virus infections. TBSMPPy also demonstrated pH‐dependent reactive oxygen species (ROS) generation under white light irradiation, endowing AIE@LD with potential photodynamic therapy (PDT) functionality. Further studies revealed that under acidic conditions, ROS production by AIE@LD significantly increased (Figure 2F), and this effect was further enhanced in the aggregated state (Figure 2G). The efficient intersystem crossing could benefit the ROS generation. It has been reported that the introduction of heteroatoms can effectively enhance intersystem crossing and is an effective strategy for designing highly efficient phosphorescent and TADF materials.^[^ ^46^, ^47^ ^]^ Based on the above results, we think effective ISC of our molecules mainly comes from the heteroatoms of the molecular skeleton. Thus, the DFT and TD‐DFT calculations were conducted to evaluate the ISC process. As shown in Table S1 (Supporting Information), the protonated TBSMPPy showed a low energy gap (∆Est = 0.181) between S1 and T1, indicated the easily intersystem crossing. Besides, the spin−orbit coupling (SOC) constants (ξ) 0.447 between S1 and T1 also reflected the smooth ISC process (Table S1, Supporting Information). This property provides excellent specificity for MPOX therapy, as AIE@LD produces substantial amounts of ROS exclusively in acidic lesion environments while generating negligible levels in normal tissues, thereby greatly increasing treatment safety. In summary, AIE@LDs exhibit superior pH responsiveness, fluorescence properties, ROS‐generating capacity, and safety, establishing a robust foundation for long‐term monitoring of MPOX and targeted wound therapy.

### 2.2 Stability of AIE@LD

**Figure 3:**
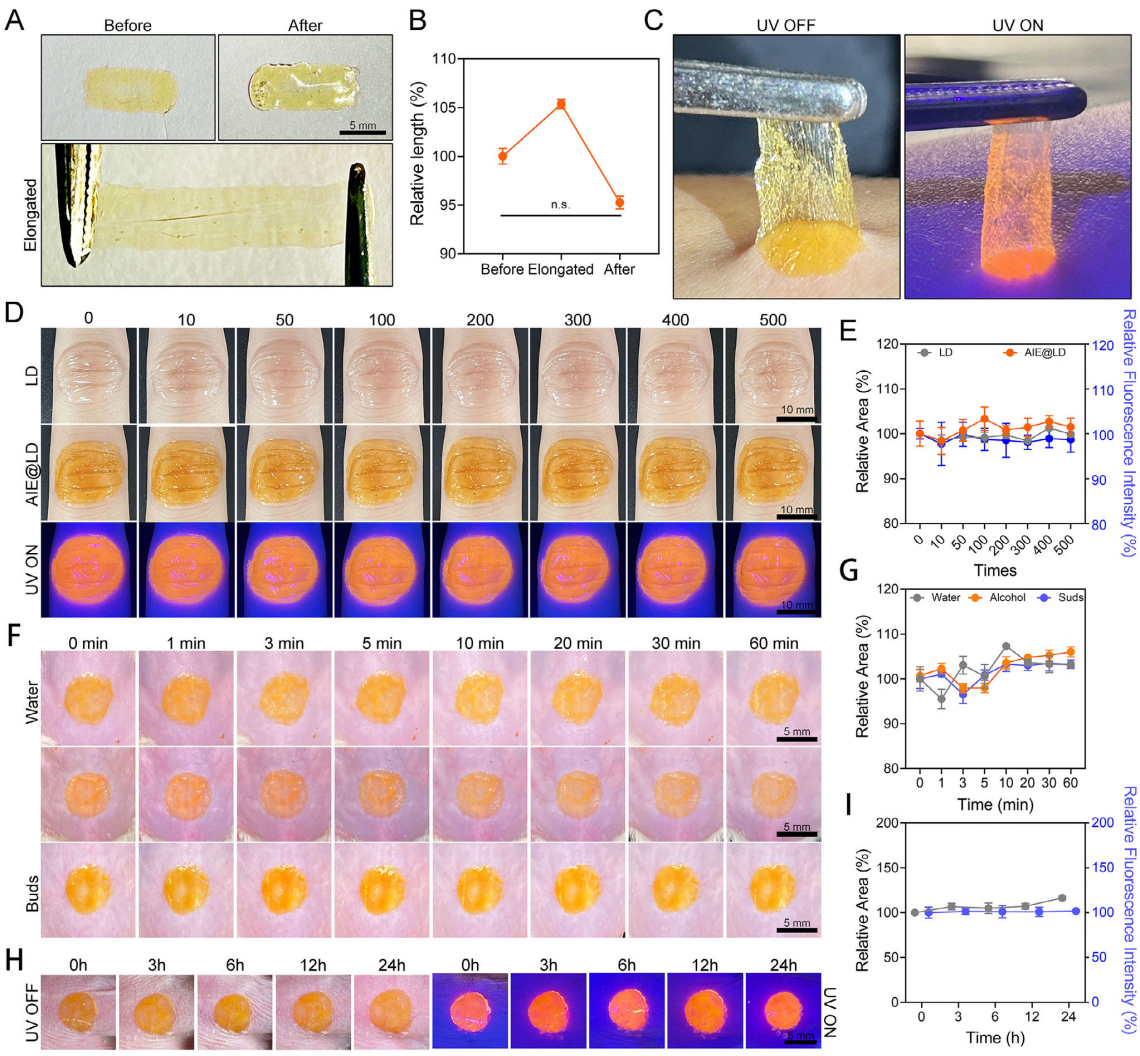
Stability of AIE@LD. A,B) Images and statistical analysis of the relative length changes of AIE@LD before, during, and after stretching (scale = 5 mm). The data are expressed as the means ± SDs (n = 3). C) Adhesion of AIE@LD to skin under white light and UV light. D,E) Images and statistical analysis of the relative area changes in the liquid dressing (LD) and AIE@LD after 0–500 flexion‐extension cycles of finger joints (scale = 10 mm). The data are expressed as the means ± SDs (n = 3). F,G) AIE@LD images and statistical analysis of the relative area change under continuous flows of pure water, 75% alcohol, and soap water over different periods (scale = 10 mm). The data are expressed as the means ± SDs (n = 3). H,I) Changes in the membrane area of AIE@LD on mouse skin over 24 h and statistical analysis (scale bar = 10 mm). The data are expressed as the means ± SDs (n = 3). ns, not significant; ^*^ *p* < 0.05, ^**^ *p* < 0.01, ^***^ *P* < 0.001.

The main characteristics of the AIE@LD dressing are its excellent flexibility, strong adhesion, and extended service life. To validate these attributes, the AIE@LD dressing was applied to a mold to form a film, which was subsequently removed via tweezers and assessed for its flexibility. Even when stretched to twice its original length with tweezers, the film reverted to its initial length and appearance (**Figure** **3**A,B), demonstrating that the AIE@LD dressing possesses remarkable elasticity and maintains significant flexibility under external stress, a critical feature for observing MPOX in skin regions located at or near joints that undergo frequent movement. To evaluate adhesion, the dressing was applied to a human arm. Lifting one edge with tweezers while applying tensile force showed that the opposite edge remained firmly attached to the skin. Under UV illumination, the dressing emitted a uniform fluorescence signal, indicating its excellent adherence and mechanical stability (Figure 3C). Flexion and extension tests were performed on the finger joints to assess the flexibility and adhesive performance of the dressing. When the finger was bent at a 90° angle, the dressing remained securely attached without visible peeling or cracking (Figure S8, Supporting Information). After 500 cycles of high‐intensity flexion and extension, AIE@LD maintained strong adhesion to the finger joint, with no significant change in area or fluorescence intensity, and its tensile strength remained comparable to that of the unmodified LD (Figure 3D,E). These findings confirm that the incorporation of TBSMPPy does not compromise the mechanical toughness of LDs while ensuring that the dressing can withstand repeated mechanical stresses in practical applications.

Additionally, the stability of AIE@LD was evaluated under various liquid conditions. Upon immersion in running water, 75% alcohol, or soapy water for 1 h, the film remained intact on the skin surface, showing no signs of detachment, coagulation, or dissolution (Figure 3F). The film area remained stable across different time points (Figure 3G), and under UV light, the dressing consistently emitted fluorescence without notable intensity loss (Figure S9, Supporting Information). These results demonstrate that AIE@LD has excellent water resistance, making it suitable for daily use. It maintains strong adhesion and effective fluorescent performance even after handwashing, disinfection, or bathing. Durability and adhesion are essential for wound dressings, as they must withstand mechanical forces from surrounding tissues and adhere reliably to wounds for effective treatment. To further evaluate its performance, we employed an active mouse model to simulate skin tension under daily movement by attaching the AIE@LD dressing to the dorsal skin of the mice and recording both morphological changes and UV fluorescence images at 3, 6, 12, and 24 h (Figure 3H,I). The results indicate that AIE@LD adhered firmly to the skin throughout the observation period, with no signs of detachment, and that the dressing area remained unchanged. Overall, these findings confirm that AIE@LD has outstanding flexibility, adhesion, and durability, allowing it to adapt to dynamic skin movements, remain stable in various aqueous environments, and reliably maintain its fluorescent signal. AIE@LD thus serves as an ideal spray film for wound protection and real‐time monitoring of wound healing.

### 2.3 In Vitro PDT Antiviral and Anti‐Inflammatory Activity

**Figure 4:**
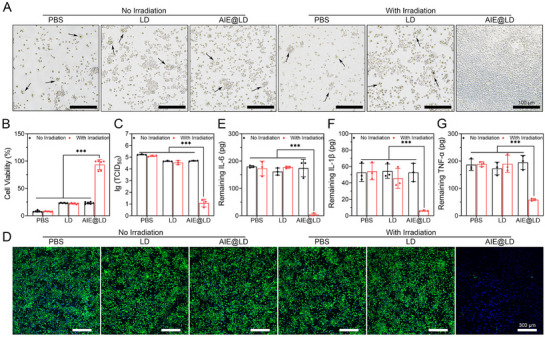
In vitro PDT's antiviral and anti‐inflammatory activity. A,B) Photos and cell viability analysis of BHK‐21 cells after different treatments (scale = 100 µm), showing the mean ± SD (n = 6). C) Evaluation of antiviral efficacy after different treatments (TCID50). The data are shown as the means ± SDs (n = 3). D) Confocal fluorescence images of GPF‐tagged variant post‐treatment (scale bar = 200 µm). Nuclei were stained with DAPI (blue); GFP‐ virus (green). E,F,G)The levels of various cytokines (IL‐6, IL‐1β, and TNF‐α) in the cell supernatants after different treatments were determined using an enzyme‐linked immunosorbent assay (ELISA). The data are shown as the means ± SDs (n = 3). The data are shown as the means ± SDs (n = 3). Data analysis was performed via one‐way ANOVA. ^*^ *p* < 0.05, ^**^ *p* < 0.01, ^***^ *p* < 0.001.

We subsequently assessed the antiviral and anti‐inflammatory properties of AIE@LD. The PBS, LD, and AIE@LD–Irradiation groups presented pronounced cytopathic effects (CPEs) and reduced cell viability, whereas cells in the AIE@LD+Irradiation group presented negligible pathological changes (**Figure** **4**A), suggesting that this treatment approach effectively protects cells from viral invasion. In addition, the CCK‐8 assay was employed to evaluate cell viability across all treatment groups, revealing that AIE@LD effectively preserved cellular activity under white light exposure, whereas cell viability in the other groups significantly decreased (Figure 4B). These results demonstrate that AIE@LD can activate the photodynamic therapy (PDT) effect under white light, efficiently eliminate active viruses, and thereby achieve a potent antiviral protective effect. To quantitatively evaluate the antiviral efficacy of each treatment modality, we employed the median tissue culture infectious dose (TCID_50_) assay to measure the quantity of remaining viable viral particles. The results indicated that the PBS, LD, and AIE@LD–Irradiation groups did not significantly reduce viral titers, whereas the AIE@LD+Irradiation group nearly eradicated the virus (Figure 4C), confirming that AIE@LD generates substantial amounts of reactive oxygen species (ROS) under white light activation, thereby achieving effective antiviral outcomes via PDT. We established a virus infection model by virus‐infected cells with GPF‐tagged variant, which was more accurately demonstrated by laser confocal microscopy to be negligible in the AIE@LD+Irradiation group, while fluorescence was abundant in the control group, indicating that the virus continued to survive in the PBS and LD groups (Figure 4D; Figure S10, Supporting Information). This highly sensitive technology fully demonstrates the advantages of AIE@LD in killing viruses.

Furthermore, an enzyme‐linked immunosorbent assay (ELISA) was conducted to measure the concentrations of the pro‐inflammatory cytokines IL‐6, IL‐1β, and TNF‐α in the culture supernatant, assessing the anti‐inflammatory effects of AIE@LD. Compared with those in the other treatment cohorts, the expression levels of these inflammatory cytokines were significantly lower in the AIE@LD+irradiation group (Figure 4E–G), indicating that this therapeutic strategy can effectively suppress virus‐induced inflammatory responses. The AIE@LD treatment strategy, when combined with white light activation, not only successfully eliminates viruses but also minimizes cellular damage and suppresses inflammation, thereby resulting in outstanding antiviral and anti‐inflammatory properties.

### 2.4 Detection of MPOX Wound Infections and Development of an Intelligent Early Warning System

**Figure 5:**
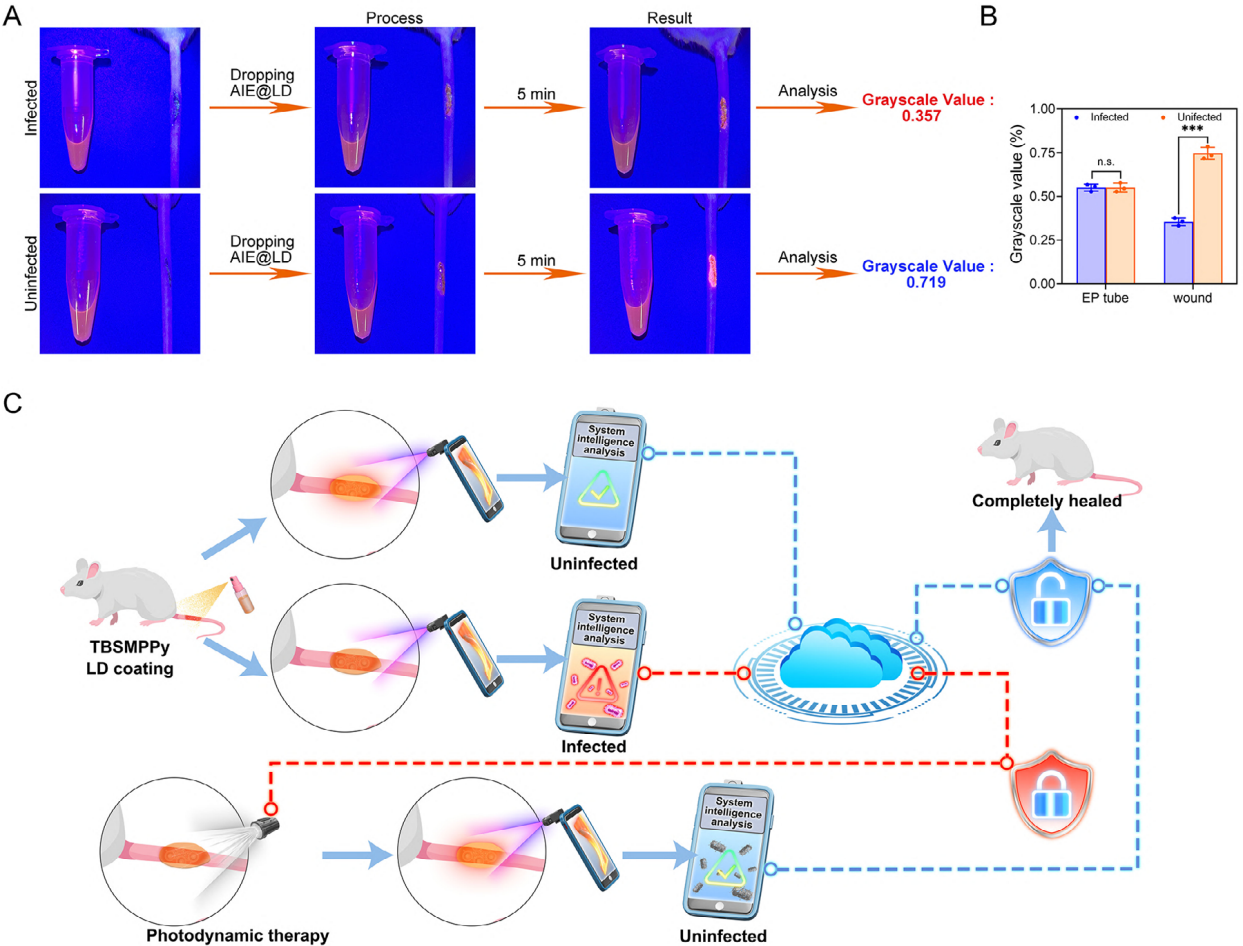
Detection of monkeypox wound infections and the construction of an intelligent, comprehensive early warning system. A,B) Quantitative analysis of images and grayscale values before and after AIE@LD detection of infected and uninfected wounds. The data are shown as the means ± SDs (n = 3). C) Schematic diagram of the intelligent early warning system for monkeypox infection monitoring and photodynamic therapy based on AIEgen. Data analysis was performed using an unpaired *t*‐test. ^*^ *p* < 0.05, ^**^ *p* < 0.01, ^***^ *p* < 0.001.

To assess the in vivo detection efficacy of AIE@LD, a scar infection model and a control group were established on the tails of the mice via the MPOX seed virus and saline, respectively, in accordance with previously reported methods.^[^ ^48^, ^49^ ^]^ The mice were divided into infected and uninfected groups, and AIE@LD was applied to the wounds, which formed a stable film within 5 min. The in vivo experimental results revealed a notable reduction in the fluorescence intensity of the wound dressing in infected mice, yielding a grayscale value of 0.357, whereas the fluorescence intensity in uninfected mice remained high, with a grayscale value of 0.719 (**Figure** **5**A). These variations in luminescence at different infection stages are attributed to the pH‐sensitive properties of the AIE molecule TBSMPPy. In uninfected wounds, the local pH is ≈6, which has a minimal impact on the fluorescence emission of TBSMPPy. In contrast, in infected environments, the pH decreases, leading to the protonation of the pyridine groups on TBSMPPy and subsequent fluorescence quenching. During the detection process, no change in fluorescence was observed in the EP tube, and the grayscale values did not differ significantly (Figure 5B), indicating that AIE@LD maintains a stable fluorescence signal unaffected by external environmental factors. These findings support the conclusion that the fluorescence changes observed in vivo are primarily due to pH alterations at the infection site rather than material degradation or interference from other environmental influences, confirming the reliability and specificity of AIE@LD as a sensor for monkeypox virus (MPXV) infection. A magnified image is shown in Figure S11A (Supporting Information), where fluorescence from the infected group significantly decreased 5 min after application, whereas fluorescence in the uninfected group remained stable. Quantitative analysis of fluorescence intensity revealed a statistically significant difference between the two groups (Figure S11B, Supporting Information), demonstrating that AIE@LD can be used to distinguish between infected and uninfected wounds with high sensitivity based on the fluorescence response, confirming its high accuracy and reliability for MPXV diagnosis.

Building upon the detection efficiency of AIE@LD, we developed an integrated system for the detection, monitoring, treatment, and prevention of MPXV. The procedure begins by attaching a UV light source to a smartphone, which is used to photograph the wound under UV illumination. The application utilizes a one‐click detection function to automatically analyze images and generate real‐time diagnostic results while simultaneously uploading data to the cloud to support informed clinical decision‐making. The full prevention and control workflow is illustrated in Figure 5C. After AIE@LD is applied to the wound, the mobile phone captures an image under UV light. The intelligent system analyzes the image in real‐time, generates a diagnostic report, and synchronizes the results with cloud storage. In the absence of infection, the dressing emits bright fluorescence, the system interface displays a green background, a health report is issued, and the data are automatically uploaded to the cloud. If an infection is detected, the fluorescence becomes faint orange, the interface turns red, an infection alert is triggered, and the results are synchronized with the disease control center to enable rapid response and containment. Simultaneously, the system recommends white‐light PDT treatment based on the test results and physician input. Following PDT therapy, infected mice are retested. If the dressing fluoresces brightly in orange, the system interprets this as successful virus elimination, deactivates the CDC alert, and confirms the progression of wound healing.

### 2.5 In Vivo Comprehensive Warning and PDT Treatment

**Figure 6:**
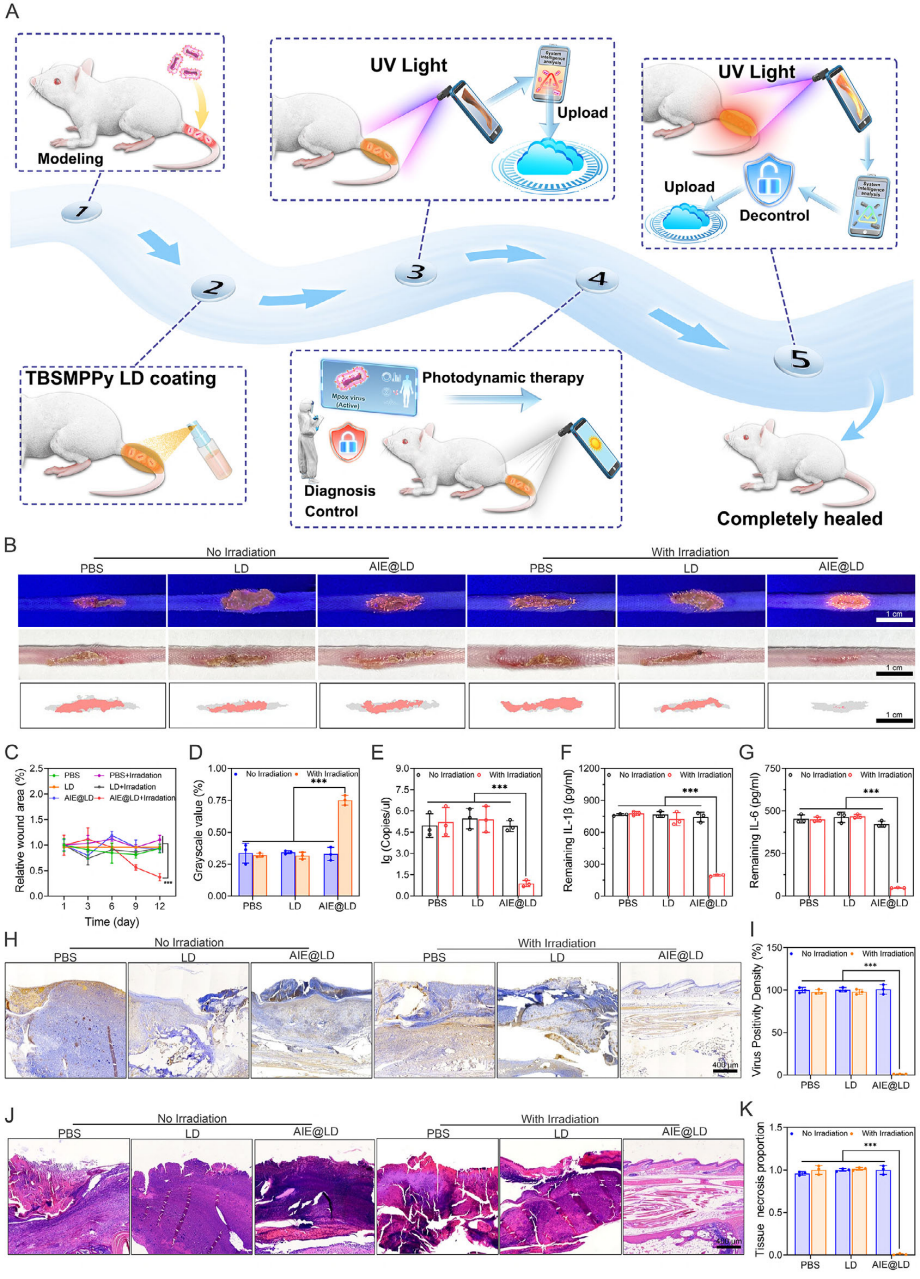
In vivo comprehensive warning and PDT treatment. A) Schematic diagram of proactive monitoring and on‐demand PDT treatment for MPOX infection. B) Photos of wounds under white light and UV light, as well as wound trajectory diagrams for different treatment groups on day 12 (scale = 10 mm). C) Quantitative analysis of the area of tail lesions in different treatment groups. D) Statistical analysis of the grayscale values of wound spray images of AIE@LD films under UV light on day 12 for different treatment groups. E) Viral load in the tail lesions of different treatment groups. F,G) Detection of pro‐inflammatory factors (IL‐1β and IL‐6) in the tail lesions of different treatment groups. H,I) Immunohistochemical staining and quantitative analysis of viral antigens in the lesion sites of different treatment groups (scale = 400 µm). J,K) Quantitative analysis of hematoxylin‐eosin (H&E) staining and injury areas in the lesion sites of different treatment groups (scale = 400 µm). The data are expressed as the means ± SDs (n = 3). Data analysis was performed via one‐way ANOVA. ^*^ *p* < 0.05, ^**^ *p* < 0.01, ^***^ *p* < 0.001.

For in vivo investigations, we employed an intelligent control system to replicate the diagnostic and therapeutic processes for MPOX infection, as illustrated in **Figure** **6**A. The MPOX vaccine virus was administered at the incision site on the mouse tail. By day six, visible lesions had formed in the infected area. AIE@LD was applied to the wound, resulting in the formation of a film that exhibited dark orange fluorescence under UV light. A mobile application was subsequently used to capture and analyze images with a single click, generating a monkeypox disease alert and synchronizing the data to the cloud. Researchers then initiated control measures in infected mice, administering white light photodynamic therapy (PDT) as needed to inhibit lesion progression while continuously monitoring treatment outcomes. After treatment, in situ detection under UV light revealed that the film emitted bright orange fluorescence. The intelligent software generated a recovery report and synchronized the results to the cloud, prompting the control center to revoke the quarantine alert.

To evaluate the efficacy of the AIE@LD‐based intelligent system in antiviral therapy, infected mice were randomly divided into six groups, each receiving a different treatment on day 6: PBS, PBS + irradiation, LD, LD + irradiation, AIE@LD, and AIE@LD + irradiation. The degree of wound healing was recorded (Figure S12, Supporting Information). In the PBS, LD, and AIE@LD‐Irradiation groups, healing was slow, with multiple pustules still evident on day 12, indicating ongoing viral replication and uncontrolled inflammation. In contrast, pustules in the AIE@LD+irradiation group rapidly formed crusts, which had detached by day 9, leading to nearly complete healing by day 12 (Figure 6B,C). On day 12, an assessment of each group revealed that only the tails of the mice in the AIE@LD+irradiation group emitted bright orange fluorescence, indicating complete viral clearance (Figure 6D). These results demonstrate that AIE@LD, in combination with white light PDT, can effectively suppress viral replication, significantly accelerate wound healing.

Previous research has suggested that AIE molecules may exert antiviral effects by obstructing viral entry, reducing replication, and preventing viral release. To further evaluate the antiviral efficacy of the different treatments, virus titers in the tail tissue were measured on day six post‐treatment. The AIE@LD+Irradiation group presented the lowest viral load, indicating the strongest antiviral effect, whereas the viral loads remained high in the other five groups (Figure 6E). Moreover, AIE‐mediated PDT has antioxidant effects on healthy tissues and cells, effectively modulating apoptosis by regulating immune cell oxidative stress responses, thereby preventing tissue damage.^[^ ^50^, ^51^ ^]^ ELISA analysis of tissue homogenates revealed significantly elevated levels of the pro‐inflammatory cytokines IL‐1β and IL‐6 in the PBS, LD, and AIE@LD‐ irradiation groups (Figure 6F,G), indicating that MPXV induces local inflammation, which results in pronounced redness, swelling, and abscess formation. In contrast, the AIE@LD + Irradiation group presented markedly reduced cytokine levels, further validating the anti‐inflammatory efficacy of AIE@LD‐mediated photodynamic therapy (PDT). Subsequently, immunohistochemical analysis was conducted on infected tail tissues (Figure 6H,I). High levels of viral antigen deposition were observed in the PBS, LD, and AIE@LD‐ irradiation groups, indicating active viral replication in these tissues.

In contrast, the AIE@LD+Irradiation group presented a significant reduction in antigen density, confirming effective viral eradication. Hematoxylin and eosin (HE) staining (Figure 6J,K) revealed extensive inflammatory cell infiltration and tissue necrosis in the PBS, LD, and AIE@LD‐irradiation groups, whereas no significant pathological damage was observed in the AIE@LD+irradiation group, underscoring the therapeutic efficacy of the treatment. Furthermore, immunohistochemical staining for TNF‐α and IFN‐γ revealed strong and diffuse nuclear expression in the PBS, LD, and AIE@LD‐irradiation groups (Figures S13 and S14, Supporting Information), indicating robust viral replication and intense inflammatory responses. These markers were significantly diminished or absent in the AIE@LD+irradiation group, supporting its antiviral and anti‐inflammatory capabilities. Masson staining corroborated these findings: the five untreated groups exhibited severe collagen fibre hyperplasia, fragmentation, and muscle fibre disorganization, whereas the AIE@LD+irradiation group presented a stable collagen quantity and morphology, with no abnormal hyperplasia (Figure S15, Supporting Information). Collectively, these results confirm that the AIE@LD‐mediated intelligent prevention and control system offers effective detection and real‐time monitoring of MPXV, precisely eliminates viruses via white light PDT, mitigates inflammatory responses, and significantly promotes wound healing, establishing a novel and intelligent approach for diagnosing and treating viral infections.

### 2.6 Blocking MPXV Transmission In Vivo through PDT with AIE@LD

**Figure 7:**
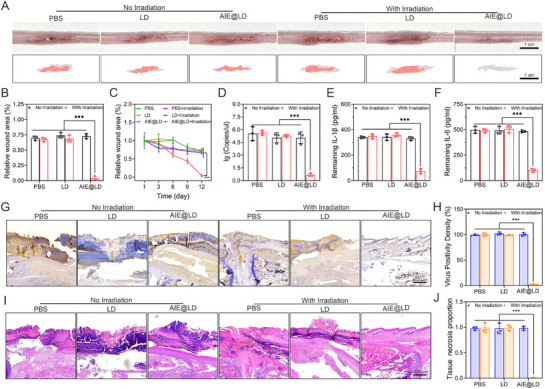
Blocking monkeypox virus transmission in vivo through PDT with AIE@LD. A) Appearance and trajectory of the wound on the tail of healthy mice 12 days after reinoculation with viral supernatant (from the tail lesions of mice treated via different methods). Scale = 10 mm, and the data are expressed as the means± standard deviations (n = 3). B,C) Statistical analysis of the wound area for each group on day 12 and at different time points. D–F) Detection of the viral load and pro‐inflammatory factors (IL‐1β and IL‐6) in tail wounds 12 days after reinoculation. G,H) After the tails of healthy mice were reinoculated with virus‐containing supernatant, immunohistochemical staining and quantitative analysis of viral antigens in the tail lesions were performed (scale bar = 400 µm). I,J) After the healthy mouse tails were reinoculated with the virus‐containing supernatant, the tail lesions were subjected to HE staining, and the necrotic areas of the lesions were quantitatively analyzed (scale = 400 µm). The data are expressed as the means ± SDs (n = 3). Data analysis was performed via one‐way ANOVA. ^*^ *p* < 0.05, ^**^ *p* < 0.01, ^***^ *p* < 0.001.

During the MPXV outbreak, the virus primarily spreads through direct contact with lesions on the skin or mucous membranes of infected individuals^[^ ^1^, ^52^ ^]^;therefore, blocking transdermal transmission is critical for effective epidemic control. To this end, we systematically evaluated the efficacy of various treatment strategies in preventing viral transmission by collecting tissue samples from the tail lesions of mice in different treatment groups and reinoculating them into healthy mice. The experimental results revealed that the mice in the PBS, LD, and AIE@LD‐irradiation groups developed viral transmission to healthy subjects (Figure S16, Supporting Information). In contrast, the mice in the AIE@LD+irradiation group presented minimal tail damage (**Figure** **7**A–C), with appearances comparable to those of the uninfected controls, suggesting that this treatment strategy effectively prevented viral spread. Moreover, viral load analysis revealed that the nonirradiated, PBS+Irradiation, and LD+Irradiation groups maintained relatively high viral titers (Figure 7D), whereas no viable virus was detected in the AIE@LD+Irradiation group. This finding demonstrates that upon activation by white light, AIE@LD can efficiently eliminate the virus, thereby disrupting the transmission route. To further validate these findings, an ELISA was conducted on affected tissues (Figure 7E,F). The PBS, LD, and AIE@LD‐irradiation groups presented significantly elevated levels of IL‐6 and IL‐1β, indicating a strong inflammatory response. Conversely, the AIE@LD+Irradiation group presented dramatically reduced inflammatory cytokine levels, likely attributable to the intelligent, on‐demand AIE‐based photodynamic therapy, which eradicates MPXV and halts its transmission, thereby preventing chronic inflammation. In addition, tail lesion tissues were subjected to immunohistochemical analysis. High levels of viral antigens were observed in the nonirradiated, PBS+irradiated, and LD+irradiated groups, whereas antigen deposition was minimal in the AIE@LD+irradiated group (Figure 7G,H), suggesting that this treatment can effectively counteract the presence of viruses and associated inflammation. Hematoxylin and eosin (H&E) staining further confirmed significant inflammatory infiltration, tissue necrosis, and skin disruption in the PBS, LD, and AIE@LD‐irradiation groups, underscoring the sustained transmissibility of the virus (Figure 7I,J).

In contrast, the tissue architecture in the AIE@LD+irradiation group remained largely intact, with no significant pathological damage observed. Furthermore, immunohistochemical staining for TNF‐α and IFN‐γ revealed strong and widespread nuclear expression in the nonirradiated, PBS+irradiation, and LD+irradiation groups (Figures S17 and S18, Supporting Information), reflecting robust inflammatory responses to MPXV infection. The AIE@LD+Irradiation group exhibited minimal inflammatory marker expression, which was likely limited to mild irritation from tail scratching. The Masson staining results (Figure S19, Supporting Information) further corroborated these findings: all groups, except the AIE@LD+irradiation group, presented pronounced collagen fibre hyperplasia and myofiber abnormalities, indicating continued viral activity and transmissibility. These findings collectively demonstrate that the AIE@LD‐based intelligent diagnostic and therapeutic system, when combined with white light irradiation, can effectively eliminate MPXV and block its transmission.

### 2.7 Biocompatibility of AIE@LD

**Figure 8:**
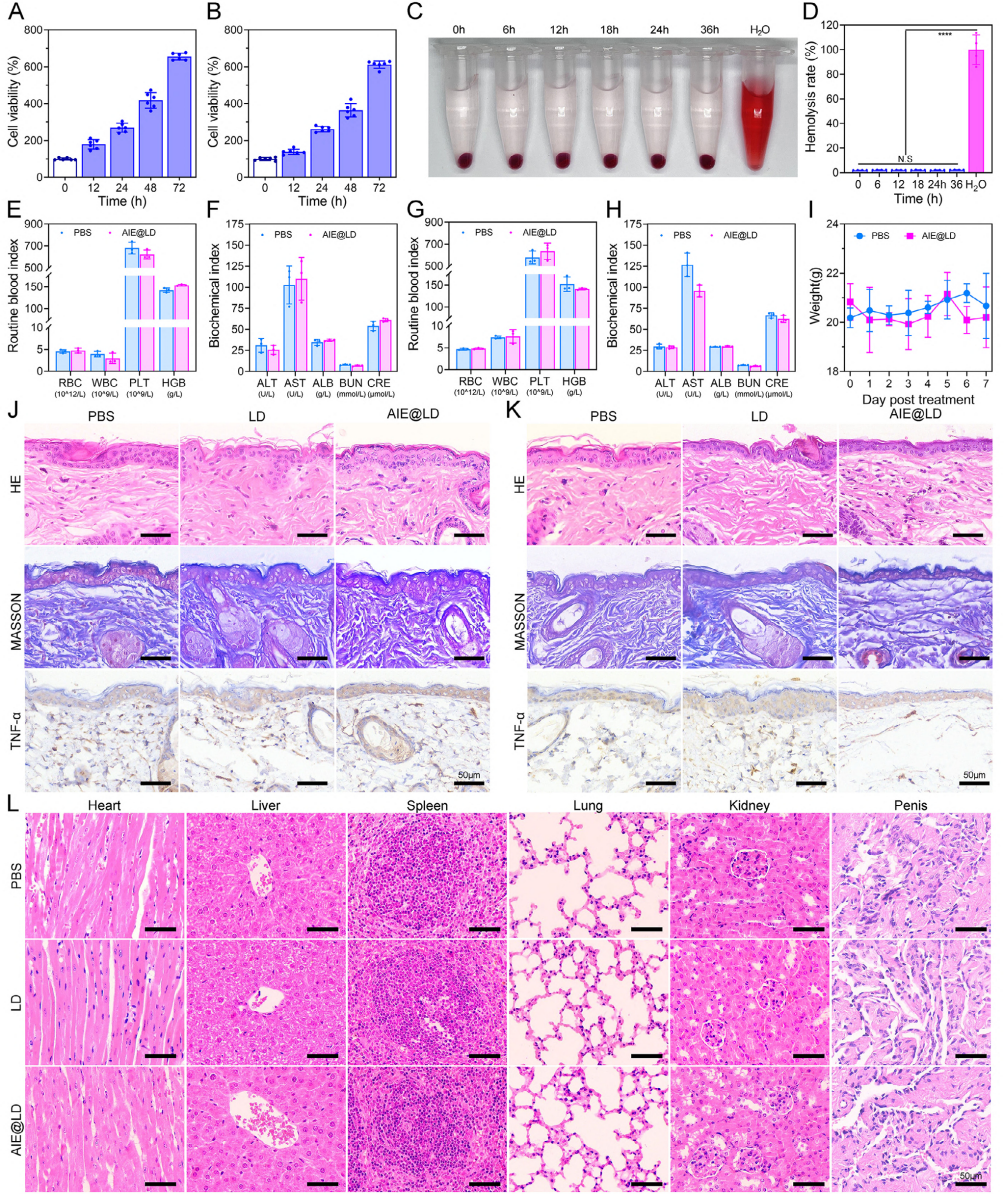
Biocompatibility of AIE@LD. A,B) Viability of healthy cells (human umbilical vein endothelial cells and L929 cells) after incubation with AIE@LD for different durations, as assessed via the Cell Counting Kit‐8. The data are shown as the means ± SDs (n = 6). C,D) Evaluation of erythrocyte lysis at different incubation times with AIE@LD, showing the mean ± SD (n = 6). E,F) Analysis of routine blood function, liver function, and kidney function on the back skin of the mice 7 days after the AIE@LD spray film was applied (mean ± SD; n = 3). G,H) Analysis of routine blood function, liver function, and kidney function in the perineal skin of mice 7 days after treatment with the AIE@LD spray film (mean ± SD; n = 3). I) The weight of the posterior skin of mice treated with different dressings was recorded, with PBS used as the control (mean ± SD; n = 3). J,K) After PBS, LD, or AIE@LD films were applied to the dorsal or perineal skin of the mice for 7 days, HE staining, Masson staining, and immunohistochemical analysis of inflammatory cytokines (TNF‐α) were performed on the mouse skin and penis (scale = 50 µm). (L) After PBS, LD, and AIE@LD were applied to the backs of healthy mice for 7 days, H&E staining was performed on vital organs (scale = 50 µm). Data analysis was performed via one‐way ANOVA. ns, not significant; ^*^ *p* < 0.05, ^**^ *p* < 0.01, ^***^ *p* < 0.001.

Excellent biocompatibility and high biosafety are fundamental prerequisites for the development of novel therapeutic agents.^[^ ^53^, ^54^, ^55^, ^56^ ^]^ We conducted a comprehensive evaluation of both the in vitro and in vivo biocompatibility of AIE@LD. As shown in **Figure** **8**A,B, coculturing AIE@LD with cells for up to 72 h and measuring cell viability at 0, 12, 24, 48, and 72 h revealed no significant inhibition of normal cell proliferation, indicating that AIE@LD has low cytotoxicity. Hemolysis assays demonstrated that coincubation with AIE@LD at a defined concentration did not induce hemolysis of red blood cells across all time points tested (0, 6, 12, 18, 24, and 36 h) (Figure 8C,D). To assess in vivo biocompatibility and simulate potential dermal toxicity, AIE@LD was topically applied to the dorsal skin of mice. Subsequent routine hematological evaluations—including red blood cell (RBC), hemoglobin (HGB), white blood cell (WBC), and platelet (PLT) counts—revealed no significant differences compared with those of the PBS‐treated controls (Figure 8E). Additionally, blood biochemical indicators such as albumin (ALB), alanine aminotransferase (ALT), aspartate aminotransferase (AST), blood urea nitrogen (BUN), and creatinine (CRE) levels remained within normal limits (Figure 8F). For the evaluation of genital toxicity, AIE@LD was applied to the penile skin of the mice. No abnormalities were detected in standard hematological or biochemical parameters (Figure 8G,H). Body weight measurements revealed no significant differences between the AIE@LD‐treated and PBS control groups throughout the study (Figure 8I). Histological assessments, including H&E staining, Masson staining, and TNF‐α immunohistochemistry, confirmed the absence of significant inflammation or tissue damage in both the skin and genital areas following 7 days of exposure to a high concentration of AIE@LD (200 µL, 30 mg kg^−1^) (Figure 8J,K). Furthermore, pathological examination of major organs, including the heart, liver, spleen, lungs, and kidneys, did not reveal any notable tissue abnormalities (Figure 8L). These findings collectively demonstrate that AIE@LD does not exhibit hematological toxicity or acute or chronic dysfunction of the hepatic or renal system. AIE@LD displays excellent biocompatibility when administered topically to both dorsal and genital skin, underscoring its potential as a safe and promising biomaterial for clinical applications.

## 3 Conclusion

To effectively prevent and manage large‐scale MPXV outbreaks, we developed an integrated smart early warning and PDT system by combining AIEgens with mobile applications. This comprehensive approach enables rapid diagnosis, real‐time monitoring, targeted photodynamic therapy (PDT) treatment, and systematic management of MPXV‐infected lesions, offering a standardized strategy for outbreak control. By leveraging the unique fluorescence characteristics and pH‐responsive behavior of the AIEgen molecule TBSMPPy, the system can accurately identify MPXV infections. Simultaneously, diagnostic data are transmitted in real‐time to a cloud‐based database, enabling efficient analysis of big data and epidemiological surveillance. Under white light irradiation, AIE@LD exhibited strong ROS generation and potent antiviral effects, effectively suppressing viral replication and spread. This intelligent early warning platform enables proactive disease management for individuals infected with MPXV, even in a home‐care setting, underscoring its practical value for widespread community deployment. Therefore, the integrated system of “portable spray, smartphone applications, and cloud databases” demonstrates significant potential in enhancing accessibility, particularly in resource‐limited environments, by providing a cost‐effective and scalable solution for disease management. Overall, this system presents a novel and promising approach for utilizing AIEgen materials in the large‐scale prevention and control of emerging infectious diseases, offering new directions for translational research and public health innovation.

## 4 Experimental Section

### Materials Synthetic Procedures

The organic photosensitizer, TBSMPPy had been previously designed and synthesized, and the synthesis in this work strictly followed the procedures reported in the previous work.^[^ ^45^ ^]^ One mol of TBSMPPy was incorporated into 1 mL of LD, and the preparation of the spray film AIE@LD was carried out. AIE@LD was successfully prepared, and its UV–vis absorption spectrum and photoluminescence (PL) spectrum were systematically characterized. To investigate the aggregation‐induced emission phenomenon, researchers studied the PL behavior under DMSO/water mixtures with different fW ratios.

### ROS Measurements

The production of ROS was examined utilizing 2,7‐dichlorofluorescein diacetate (DCFH‐DA) as a probe. DCFH‐DA was converted to 2,7‐dichlorofluorescin (DCFH) under alkaline conditions. Subsequently, DCFH was converted into the extremely fluorescent 2,7‐dichloro fluorescein (DCF) in the presence of reactive oxygen species (ROS). The excitation wavelength for DCF was 488 nm, with emission at 525 nm. The quantum yield of DCF was 90 percent. Compounds were added to the activated DCFH solutions at doses of 40 and 2 µm, respectively. The solutions were subjected to white light at an intensity of 5 mW cm^−2^ for a period of 5 min. The emission intensity of the DCF solution at 525 nm was recorded every 20 s with an excitation wavelength of 488 nm.

### AIE@LD Stability Test

To assess the flexibility and mechanical stability of the sprayed film AIE@LD, it was sprayed onto a mold surface to form a thin film. After drying, the film was removed with tweezers and stretched to twice its original length to observe its elasticity. In the adhesion test, the film was applied to the skin surface of the forearm. One edge was lifted with tweezers, and tension was applied to record whether the film remains adhered on the other side. Flexibility testing involves attaching the film to the finger joint, performing 90° flexion and extension movements, and repeating 500 high‐intensity bending cycles. The adhesion status and area changes of the film layer were observed, and fluorescent images were captured under UV light to assess its stability. In the liquid resistance test, the membrane‐adhered skin was sequentially immersed in flowing water, 75% alcohol, and soapy water for 1 h each, and it was recorded whether the membrane layer peels off, dissolves, or experiences fluorescence decay. In animal model testing, the AIE@LD membrane was applied to the dorsal skin of mice. Under free‐moving conditions, morphological and fluorescent images were captured at 3, 6, 12, and 24 h to assess their adhesion durability and signal stability on a dynamic skin surface.

### Cell Culture and Virus

The BHK‐21 kidney cell line (CSTR:19375.09.3101HAMSCSP5287) from young Syrian hamsters, the RAW 264.7 murine macrophage cell line (CSTR:19375.09.3101MOUSCSP5036), the L929 mouse epithelioid fibroblast cell line (CSTR:19375.09.3101MOUGNM28), and the human umbilical vein endothelial cell (HUVEC, CSTR:19375.09.3101HUMSCSP5330) line were acquired from the National Collection of Authenticated Cell Cultures. All cells were confirmed to be free of mycoplasma and other pathogens. All cells were grown in RPMI 1640 media or DMEM enriched with 10% FBS and 1% penicillin‐streptomycin at 37 °C in a humidified environment containing 5% CO_2_. The vaccinia virus strain Tian Tan and the GPF‐tagged variant were cultured and titrated on BHK‐21 cell monolayers. All investigations with the vaccinia virus were conducted in a biosafety level II facility.

### TCID50

The median tissue culture infectious dose (TCID_50_) assay was performed to evaluate the antiviral efficacy of different treatments. BHK‐21 cells were seeded into 96‐well plates at a density of 5000 cells per well and incubated overnight at 37 °C with 5% CO_2_ to allow monolayer formation. Vaccinia virus (VACV) samples were collected from six groups: PBS, LD, and AIE@LD with or without white light irradiation. Each viral sample was serially diluted twofold across 14 wells using DMEM as the diluent. From each dilution, 35 µL was added to five replicate wells of the cell plate, while control wells received no virus. After incubation at 37 °C for 1 h to allow viral adsorption, 180 µL of DMEM supplemented with 10% fetal bovine serum (FBS) was added to each well. Cytopathic effects were evaluated on day 4 post‐infection, and TCID_50_ values were calculated using the Reed–Muench method.

### ELISA Assay

The enzyme‐linked immunosorbent assay (ELISA) was used to quantitatively detect the levels of target proteins in the samples. Following the instructions in the kit manual (manufacturer: MIbio, catalog number: ML2133), samples or standards were added to a 96‐well plate pre‐coated with specific antibodies, with 100 µL added to each well, and incubated at 37 °C for 1 h. Remove the liquid, wash the plate three times with wash buffer (300 µL per wash). Add the enzyme‐labeled secondary antibody (HRP‐labeled) at 100 µL per well, incubate at 37 °C for another hour, and repeat the washing steps. Add 100 µL of the color development substrate solution (TMB) to each well, incubate at room temperature in the dark for 15 min, and add 50 µL of stop solution to terminate the reaction. Measure the absorbance (OD value) of each well at 450 nm using an enzyme‐linked immunosorbent assay reader. Plot a standard curve using the standard samples and calculate the concentration of the target factor in the samples.

### Establishing a Rash Replacement Model following MPXV Infection

The Experimental Animal Ethics Committee of the Experimental Animal Center of Southern Medical University evaluated and approved all animal research. Eight‐week‐old male Balb/c mice were obtained from the Laboratory Animal Center at Southern Medical University. The animal experiments related to the Vaccinia virus were performed in the SL‐2 Laboratory in Guangdong, recognized as the Guangdong Provincial Key Laboratory of Tropical Disease Research. Mice were initially anesthetized by intraperitoneal injection. A 20 µL solution of vaccinia virus (5 × 10^6 pfu) was subsequently administered to the tail, ≈1 cm from the base. The skin was subsequently scraped 20 times with a 1 cm long syringe needle (29G), facilitating the entry of the virus into the abrasion. Following a week, the researchers examined the tail lesions of the mice. The successful implementation of the mouse tail scratch model (MPOX rash substitute model) was validated by the appearance of yellow scabs and rash‐like lesions.

### The Establishment of an Advanced Early Warning System

This research has established an advanced infection early warning system utilizing smartphones. The system incorporates a UV light source within the smartphone and utilizes the device's camera to capture images of wounds under the UV light spectrum. The system utilizes a one‐click detection program to automatically analyze images, produce real‐time detection results, and upload data to the cloud to facilitate clinical decision‐making.

The monkeypox seedling virus (infection group) and saline (control group) were inoculated into the scratch model on the mouse tail to establish a scar infection model. Subsequently, AIE@LD was applied to the wound surface, creating a stable self‐luminescent film within 5 min. Wound images were captured using a smartphone under UV light, and the system autonomously analyzed the image features and produced a diagnostic report. The test results were concurrently uploaded to the cloud.

In the absence of infection, the dressing exhibits a vivid fluorescence, the system interface presents a green background, and a health report was produced, with pertinent data automatically synchronized to the cloud. Upon detection of an infection, the dressing exhibits a subtle orange fluorescence, the system interface illuminates red, an infection alert was promptly issued, and the data was synchronized with the disease control center to enable timely intervention and mitigate the spread of the epidemic.

Furthermore, the system will adeptly suggest PDT in accordance with the test outcomes and medical protocols. Post‐PDT treatment, imaging and analysis will be performed once more on the infected mice. Upon detection of a bright orange fluorescence in the dressing, the system will ascertain that the virus has been eradicated, deactivate the CDC alarm, and document the healing outcomes, thereby ensuring accurate treatment of the infected region and visual oversight of the healing progression.

### In Vivo Elimination of Virus and Prevention of Disease Transmission

According to the specified criteria, 30 mice were randomly chosen and evenly allocated into six groups. A tail‐scratch mouse model was developed using the aforementioned method. Six days later, six cohorts of mice will receive treatment with PBS, LD, and AIE@LD on the tail skin lesion site, with each treatment comprising both illuminated and non‐illuminated subgroups, each constituting fifty percent of the total. Images of the tail lesion regions for each group will be captured every three days, with ongoing monitoring for a duration of two weeks. Following the euthanasia of the mice via cervical dislocation, tail tissues were harvested from each group. Viral titers and necrosis levels at the lesion sites were assessed using quantitative reverse transcription polymerase chain reaction (qRT‐PCR), immunohistochemistry, and HE staining. The magnitude of damage and antigen density were quantified utilizing ImageJ software (National Institutes of Health, Bethesda, MD, USA) to facilitate a more precise analysis of the results. To further assess in vivo antiviral and anti‐inflammatory activity, the collected tail specimens were isolated, pulverized, and re‐suspended in 1000 µL of 1× PBS, homogenized with a refrigerated grinder (LUKYM‐I, 70 Hz, 5 min). The samples were subsequently centrifuged at 12 000 rpm for 5 min, and the supernatant was extracted. Vaccinia virus concentrations were quantified using qRT‐PCR. The primer sequences for qPCR were: vaccinia virus forward, 5′‐ACATCTGGAGAATCCACAACA‐3′; vaccinia virus reverse, 5′‐CATCATCGGTGGTTGATTTA‐3′; vaccinia virus probe, 5′‐FAM‐GAGACTCCGGAACCAAT‐TAMRA‐3′. The concentrations of pro‐inflammatory cytokines in each group were assessed using precoated ELISA kits for mouse IL‐1βand IL‐6 to evaluate the in vivo anti‐inflammatory efficacy of various treatment strategies. Immunohistochemistry and hematoxylin‐eosin staining were employed to visually assess viral antigen density and tissue necrosis levels, respectively. Image J was utilized to compute the scores. Thereafter, to assess the impact of various treatment strategies on inhibiting virus transmission in vivo, equal volumes of the collected samples from each group were combined with 1 mL of 1× PBS, subsequently homogenized in a frozen grinder (LUKYM‐I, 70 Hz, 5 min), and centrifuged at 12 000 rpm for 5 min to isolate the supernatant. The virus was introduced into abrasions on the tails of healthy mice, and these abrasions were monitored and documented with a camera over a period of 12 days. The outcomes were evaluated utilizing ImageJ software and GraphPad Prism 7.0 (GraphPad, San Diego, CA, USA). The pro‐inflammatory cytokines INF‐γ, IL‐6, and IL‐1β were measured utilizing ELISA kits. Immunohistochemical staining, hematoxylin and eosin staining, and Masson's trichrome staining were employed to quantify viral particles and inflammatory factors in the collected tail‐diseased tissues, as well as to assess the density of viral antigens and the extent of tissue necrosis. Image J was employed to compute the scores.

### Biosafety Assessment

To evaluate the biocompatibility of different treatments, PBS, LD, and AIE@LD, with or without irradiation, were administered to BHK‐21 cells for 72 h. The proliferation and viability of the cells were then assessed utilizing the CCK‐8 kit supplied by Beyotime Biotechnology. The hemolysis experiment was concurrently conducted on individuals exposed to a specified concentration of AIE@LD. They were under continuous observation for 36 h, during which hemolysis rates were documented at 0, 6, 12, 18, 24, and 36 h. To assess toxicity in living creatures, namely healthy female Balb/c mice aged 8 weeks, the mice were administered PBS, LD, and AIE@LD, with or without irradiation, over a period of 12 days. The mice's body weight was meticulously monitored and documented throughout the trial. On the twelfth day, venous blood samples were collected to assess liver function indicators including ALB, AST, and ALT, alongside renal function indicators such as CRE and blood urea nitrogen BUN. Subsequently, the skin and penises were collected from each group of mice for histological analysis and cytokine detection.

### Statistical Analysis

The results were expressed as the mean ± standard deviation (SD) derived from at least three different experiments. Group disparities were assessed utilizing an unpaired *t*‐test and one‐way analysis of variance (ANOVA), succeeded by Tukey's post‐hoc test (GraphPad Prism 7.0). The threshold for statistical significance was established at *p* < 0.05.

## Conflict of Interest

The authors declare no conflict of interest.

## Author Contributions

W.W., Z.L., M.L., J.Z. and R.Z. contributed equally to this work. K.Z., Z.Z., C.S. and Y.H. conceived the study and supervised the project. W.W., Z.L., M.L., J.Z. and R.Z. designed the experiments and performed most in vitro and in vivo experiments with the contribution of K.L., X.L., S.W., J.Y., M.P. and F.M. W.W., Z.L., M.L., J.Z. and R.Z. wrote the manuscript. All authors commented on the manuscript.

## Supporting information



Supporting Information

## Data Availability

The data that support the findings of this study are available from the corresponding author upon reasonable request.
