# Supplementary material for: AIEgen‐Based Proactive Early Warning System and Precise Treatment Strategy for Monkeypox Prevention and Control
Source: Adv Sci (Weinh). 2025 Dec 5;13(9):e15865. doi: 10.1002/advs.202515865 (PMC12904085; doi:10.1002/advs.202515865)
Supplement: Supplementary file 1 — Supporting Information [file ADVS-13-e15865-s001.docx]

**Supplementary Information**

AIEgen-based Proactive Early Warning System and Precise Treatment Strategy for Monkeypox Prevention and Control

Wei Wang^1,2†^, Zining Liu^1,3†^, Mengjun Li^3†^, Judun Zheng^1†^, Ruilin Zhang^1†^, Ke Liu^3^, Xiaoxue Li^1^, Shaoying Wen^2^, Jingze Yu^2^, Meijia Pan^2^, Fengbo Ma^2^, Kun Zhou^4*^, Zheng Zhao^4^*, Chenguang Shen ^3,5*^, Yuhui Liao ^1,2*^

**Synthesis of TBSMPPy**

The TBSMPPy was reported and provided by our collaborator，and synthesized according to literature reports.^[1]^

4-(7-bromo-5,6-difluorobenzo[c][1,2,5]thiadiazol-4-yl)-N,N-diphenylaniline (1.0 g, 2.02 mmol), (4-(pyridin-4-yl)phenyl)boronic acid (0.4 g, 2.02 mmol), K2CO3 (2.23 g, 16 mmol) and Pd(PPh3)4 (70.03 mg, 0.06 mmol) were added into a 500 mL two-necked roundbottom flask with 150 mL of distilled toluene and 50 mL water. The mixture was then heated to reflux under nitrogen for 10 h. Cooling to room temperature, the mixture was poured into water and extracted with DCM three times. The organic phase was washed with saturated NaCl solution and dried with anhydrous magnesium sulfate. After filtration and solvent evaporation, the residue was purified by silica-gel column chromatography using EA/PE mixture (1/10, v/v) as eluent. Yellow solid was obtained. Subsequently, Sodium hydride (2 mmol) was dissolved in the dry DMF solution and then dropped into the malononitrile (1 mmol) at 0 oC for 30 min. Carbon disulfide (2 mmol) was added and reacted for 2 hours at room temperature. Then above afford yellow solid (113 mg, 0.2 mmol) was dissolved in the dry DMF and dropped into the mixture. The mixture was allowed to stir at 70 °C overnight. Upon cooling to room temperature, the mixture was poured into water and extracted with DCM three times. The organic phase was washed with saturated NaCl solution and dried with anhydrous magnesium sulfate. After filtration and solvent evaporation, the residue was purified by silica-gel column chromatography using EA/DCM mixture (1/8, v/v) as eluent. Orange solid of product TBSMPPy was obtained in a yield of 40% (268.33 mg). 1H NMR (400 MHz, CDCl3): δ 8.78 (d, 2H, J = 4.52 Hz), 7.93-7.80 (m, 4H), 7.79-7.76 (m, 2H), 7.63-7.58 (m, 2H), 7.38-7.33 (m, 5H), 7.28-7.26 (m, 3H), 7.25-7.20 (m, 2H), 7.16-7.11 (m, 4H). 13C NMR (100 MHz, CDCl3): δ 153.1, 146.9, 146.8,146.7, 146.6, 130.6, 130.4, 130.3, 130.2, 130.0, 129.9, 129.8, 129.7, 129.6, 129.5, 128.0, 126.0, 125.9, 125.8, 125.7, 124.5, 124.4, 124.3, 124.1, 122.5, 122.3, 121.1, 120.9, 77.3, 77.2, 77.0, 76.7. There was no 19F NMR signal. HRMS (ESI) m/z: [M+H]+ calcd for C39H23N6S3 671.1146; found 671.1140.

**Scheme S1**. Synthetic route of TBSMPPy. Reproduced with permission from Ref.^[1]^ Copyright 2023 Elsevier Inc.


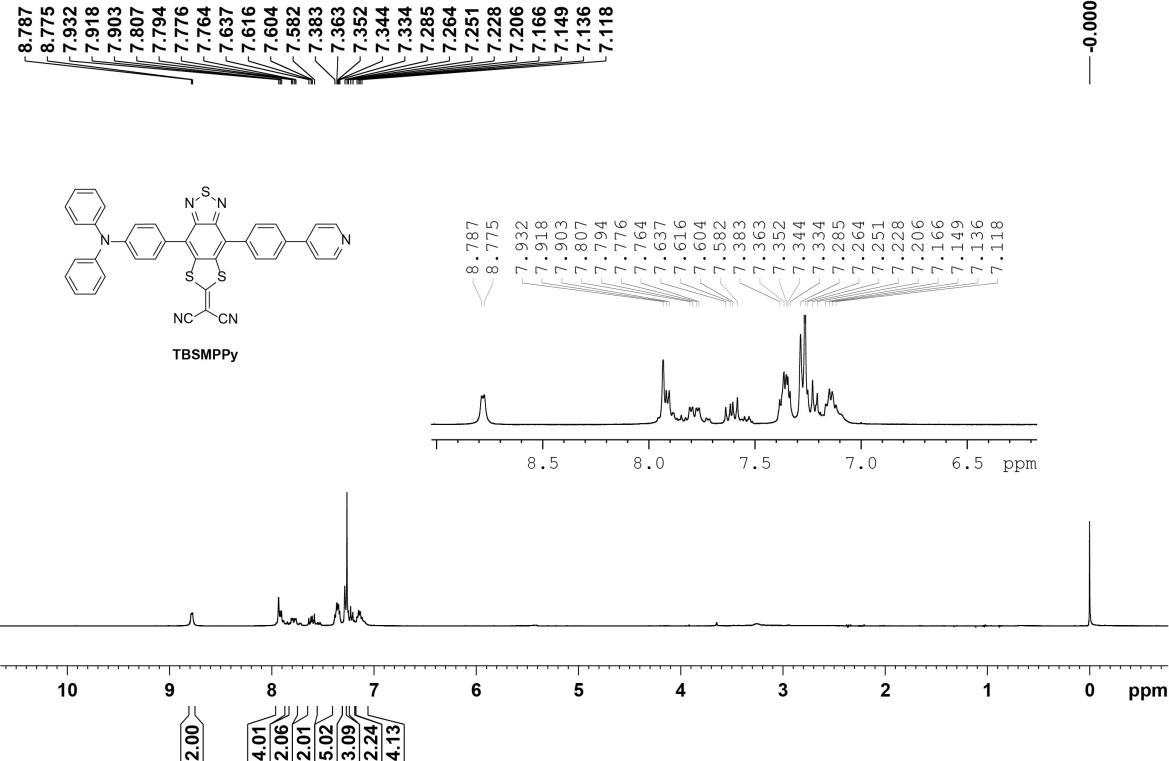


**Figure S1.** ^1^H NMR (CDCl_3_, 400 MHz) spectra of TBSMPPy.


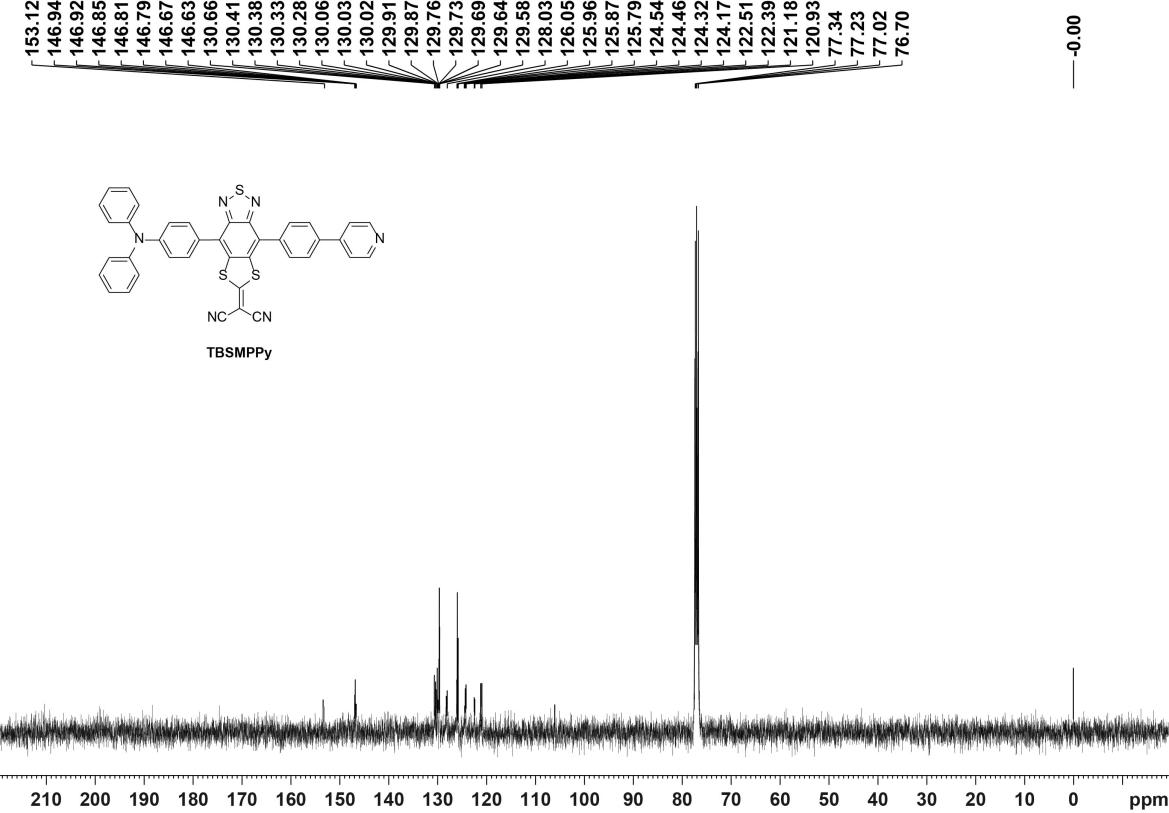


**Figure S2.** ^13^C NMR (CDCl_3_, 100 MHz) spectra of TBSMPPy.


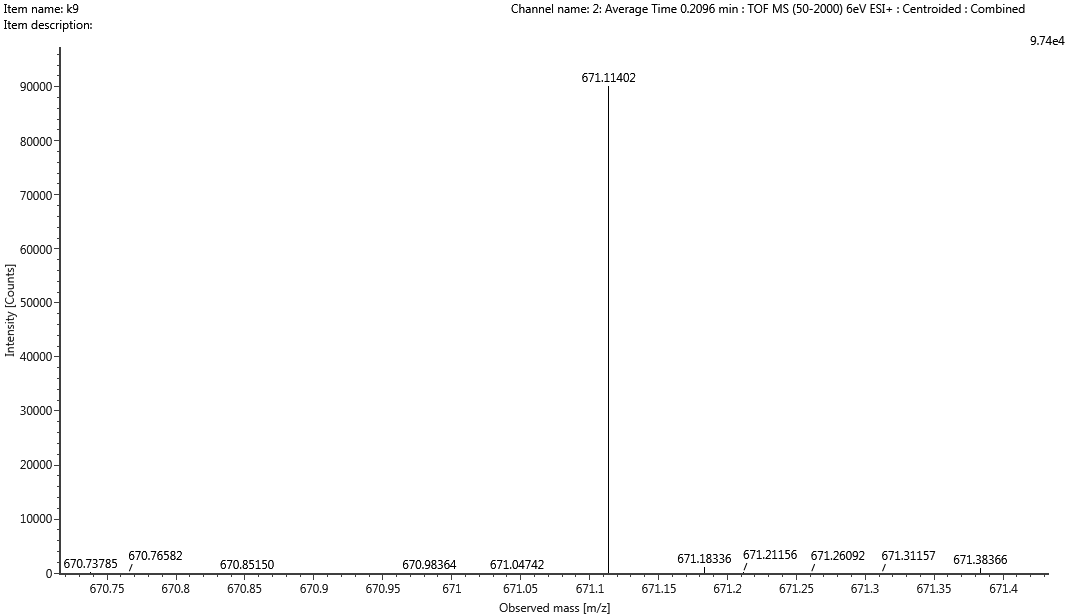


**Figure S3.** HR-MS spectra of TBSMPPy.


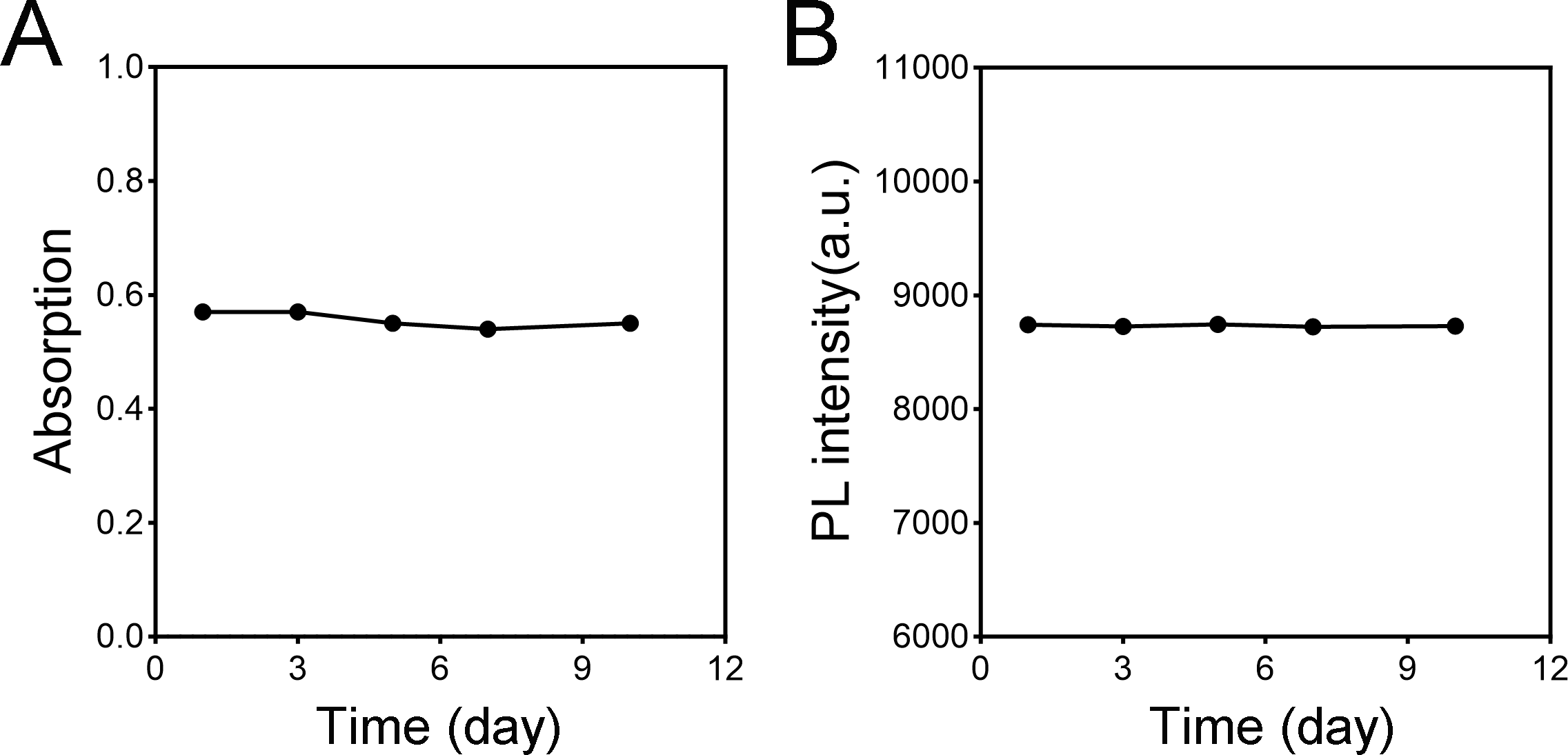


**Figure S4**. The biostability of TBSMPPy (a) absorption, (b) PL intensity of TBSMPPy dispersed in PBS with 10 % FBS at 37 °C at different times.


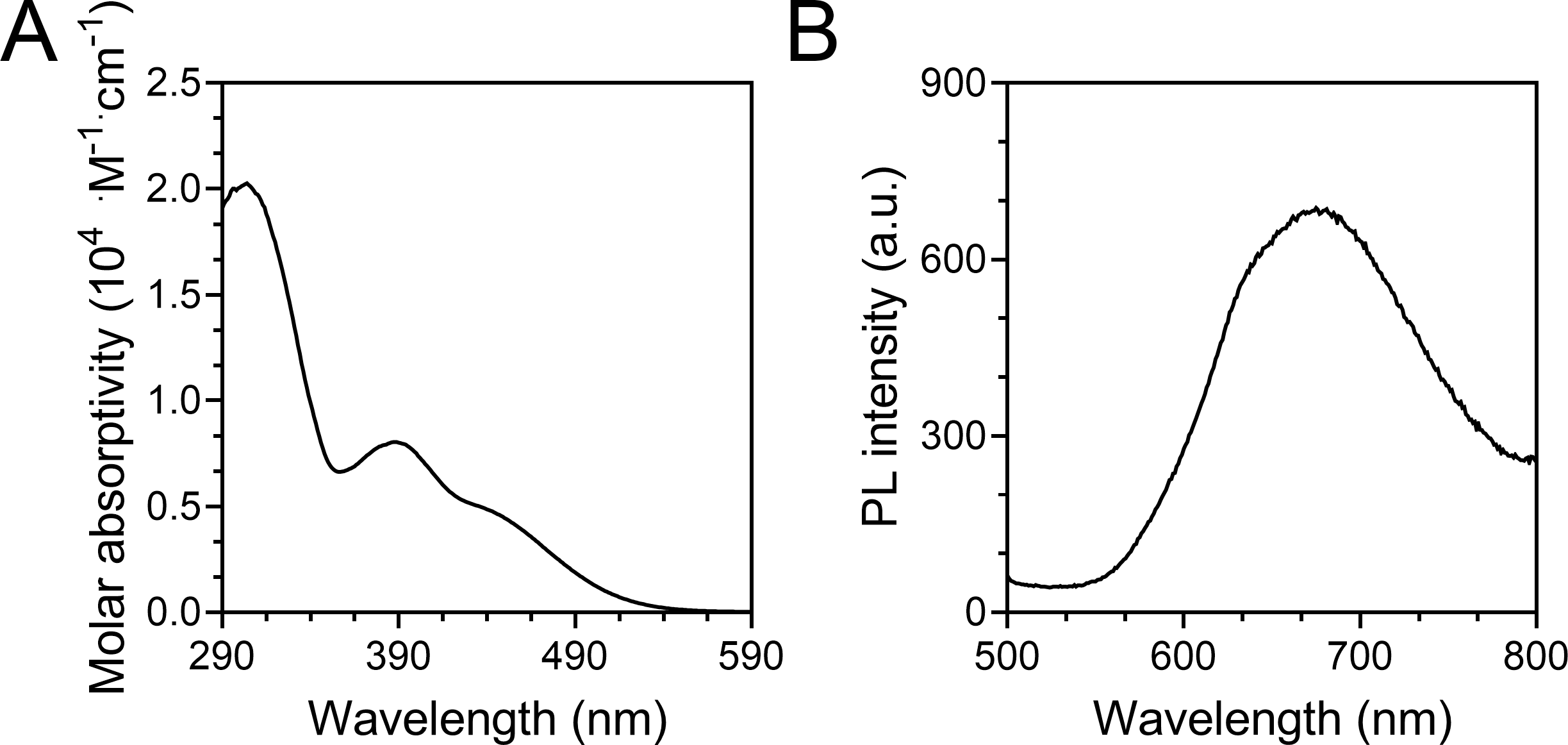


**Figure S5.** (A) UV-Vis absorption spectrum and photoluminescence (PL, B) spectrum of TBSMPPy in dimethyl sulfoxide (DMSO).


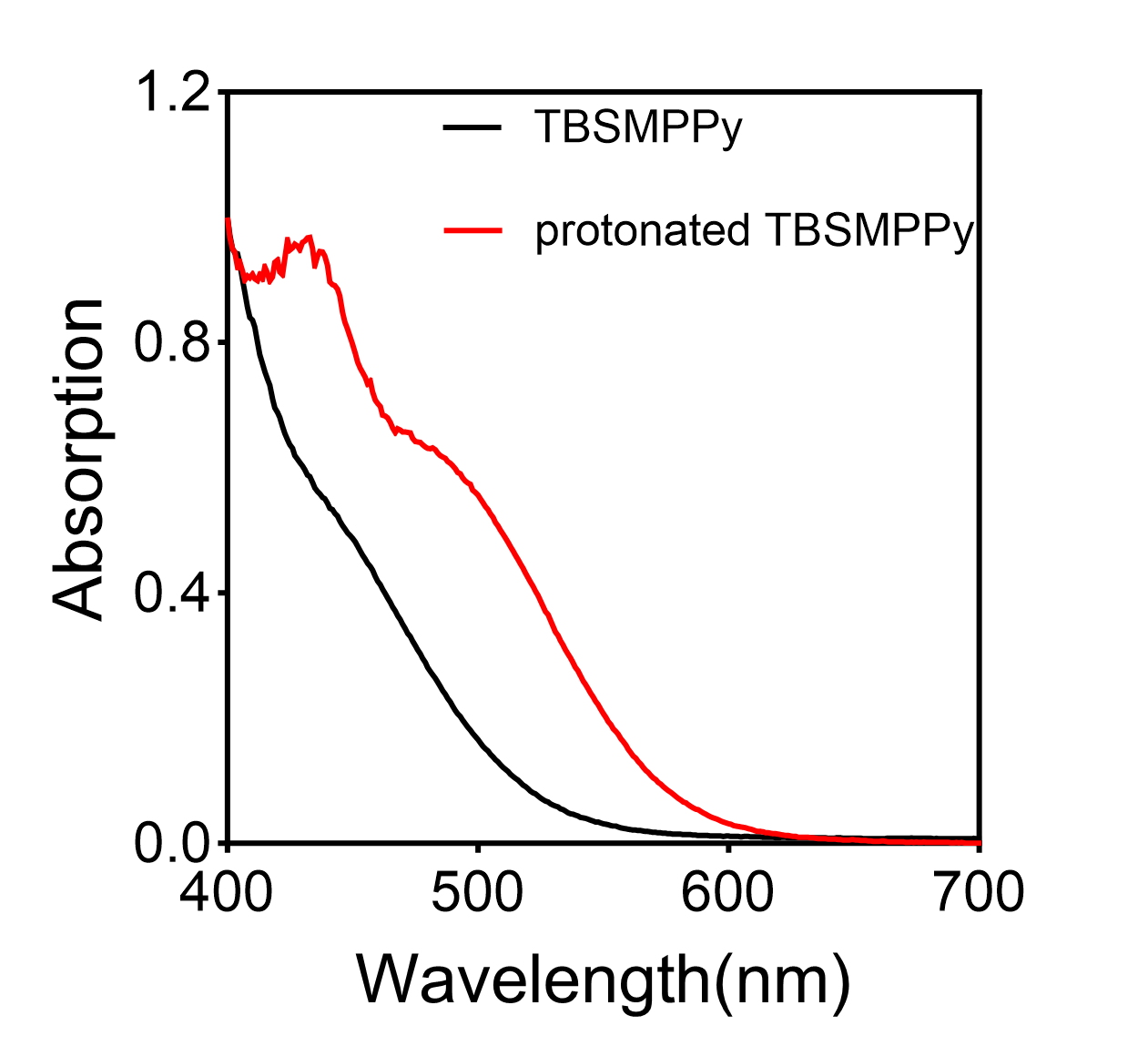


**Figure S6.** The UV spectra of TBSMPPy before and after protonation.


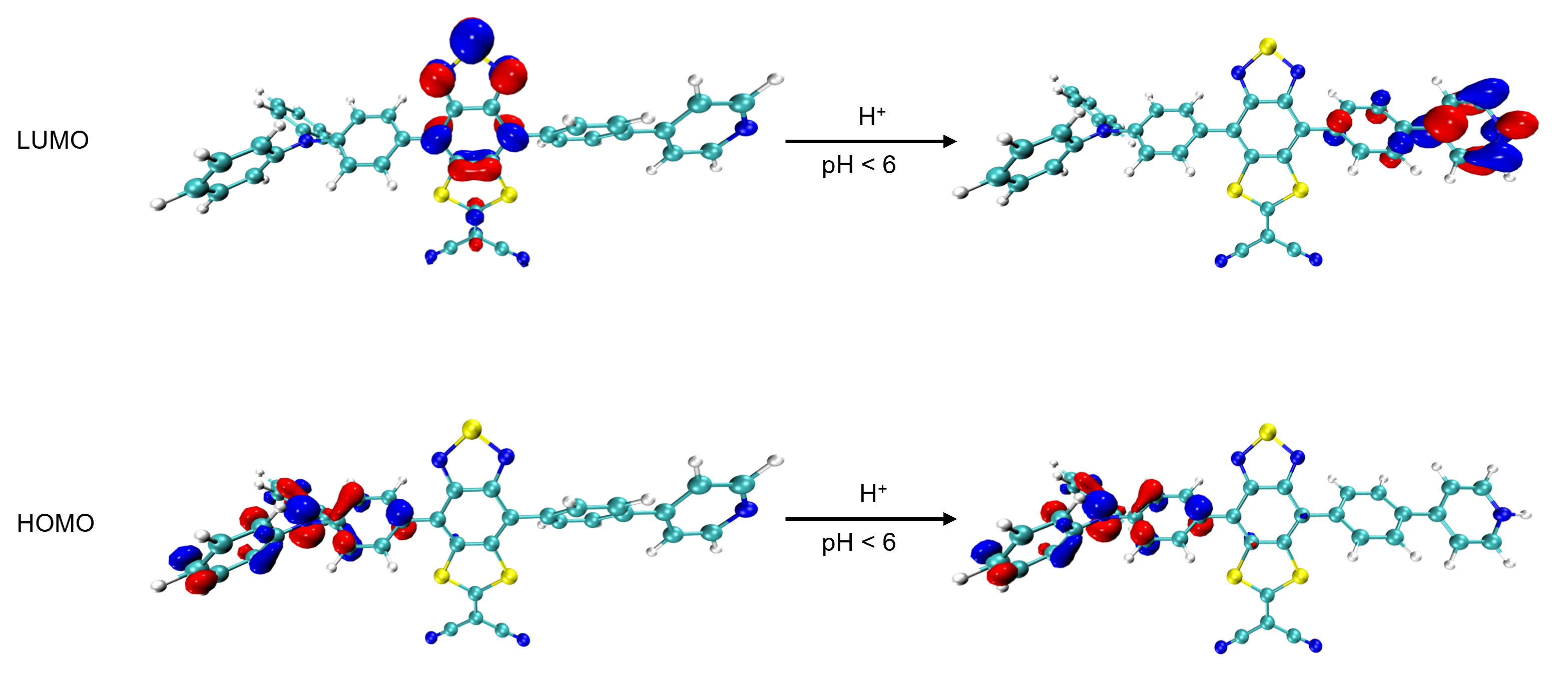


**Figure S7**. The HOMO and LUMO orbitals of TBSMPPy and protonated TBSMPPy. Reproduced with permission from Ref. ^[1]^ Copyright 2023 Elsevier Inc.


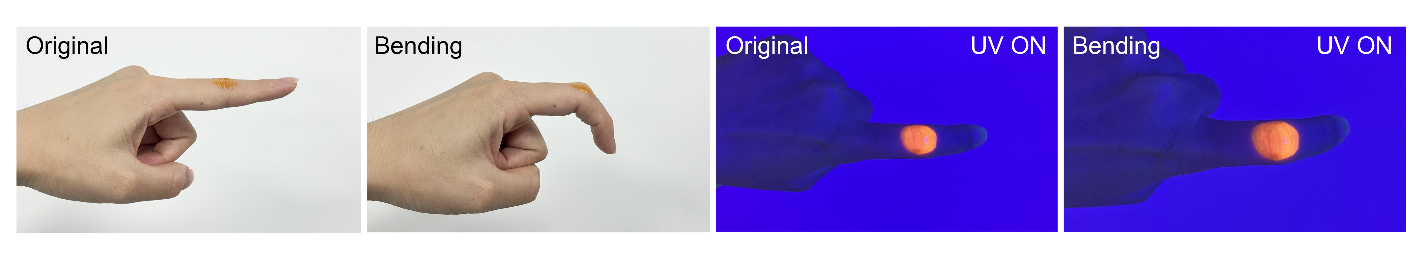


**Figure S8**. AIE@LD adheres to the skin in its initial condition and flexes under white and ultraviolet (UV) light.


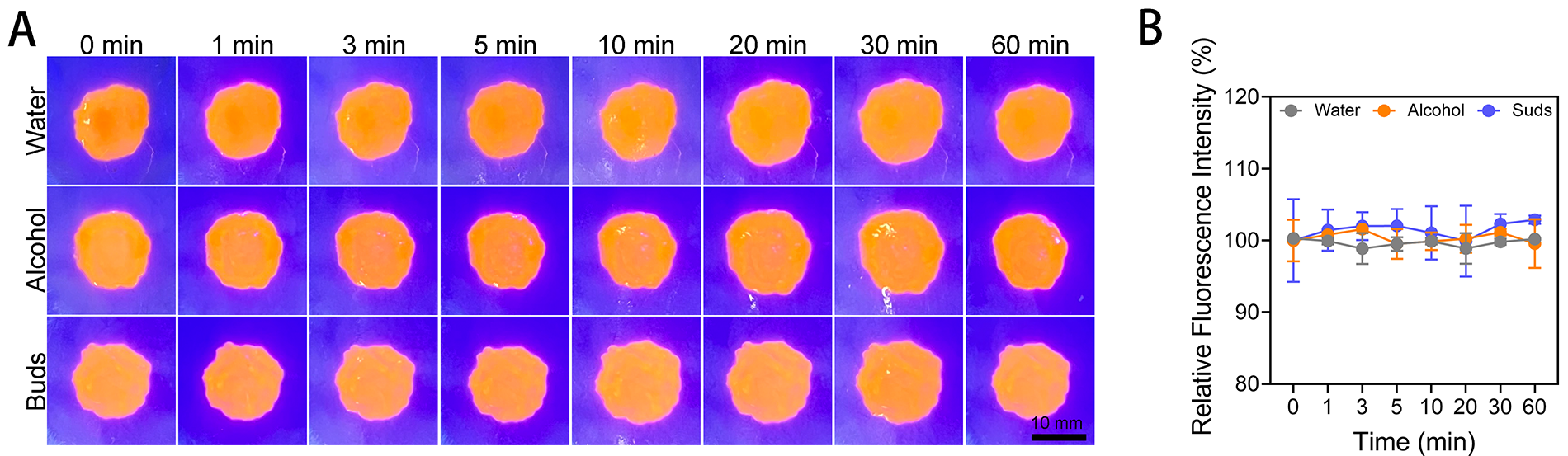


**Figure S9.** (A) AIE@LD was sprayed on the mouse skin, and after being continuously exposed to flowing pure water, 75% alcohol, and soapy water for different durations, fluorescence images under UV light and quantitative analysis of the relative fluorescence normalized grayscale values (B).


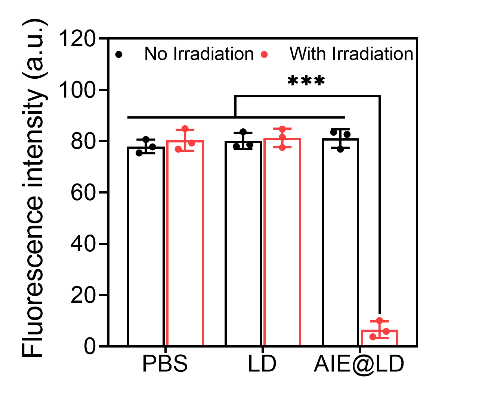


**Figure S10.** Quantitative analysis of confocal images of GFP- virus after different treatments. The data are shown as mean ± SDs (n = 3). Data analysis was performed using one-way ANOVA. **p* < 0.05, ***p* < 0.01, ****p* < 0.001.


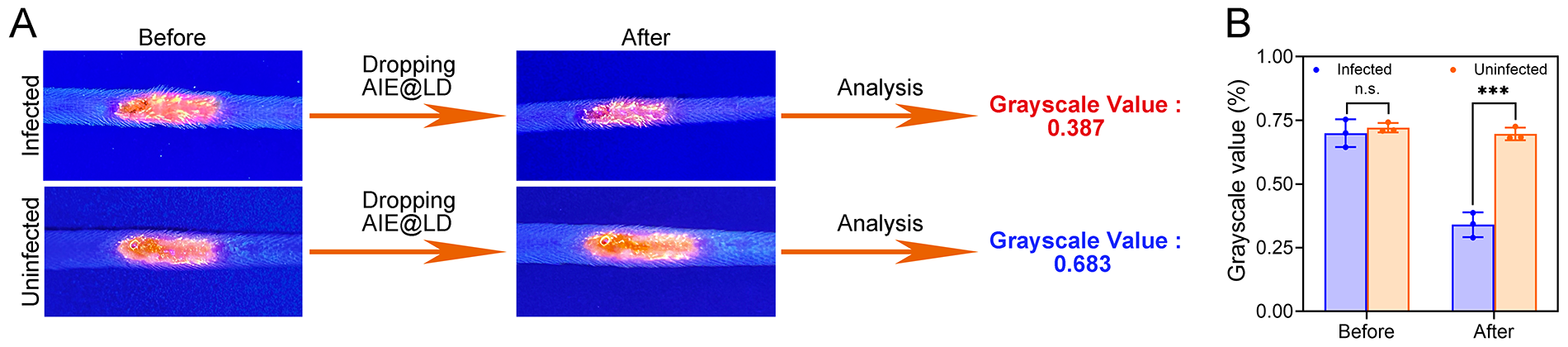


**Figure S11.** **AIE@LD response to infected and non-infected wounds.** (A) Infection and non-infection groups were analyzed with magnified images and grayscale value quantification immediately after adding AIE@LD and 5 minutes post-addition (B). The data are shown as mean ± SDs (n = 3). Data analysis was performed using one-way ANOVA. **p* < 0.05, ***p* < 0.01, ****p* < 0.001.


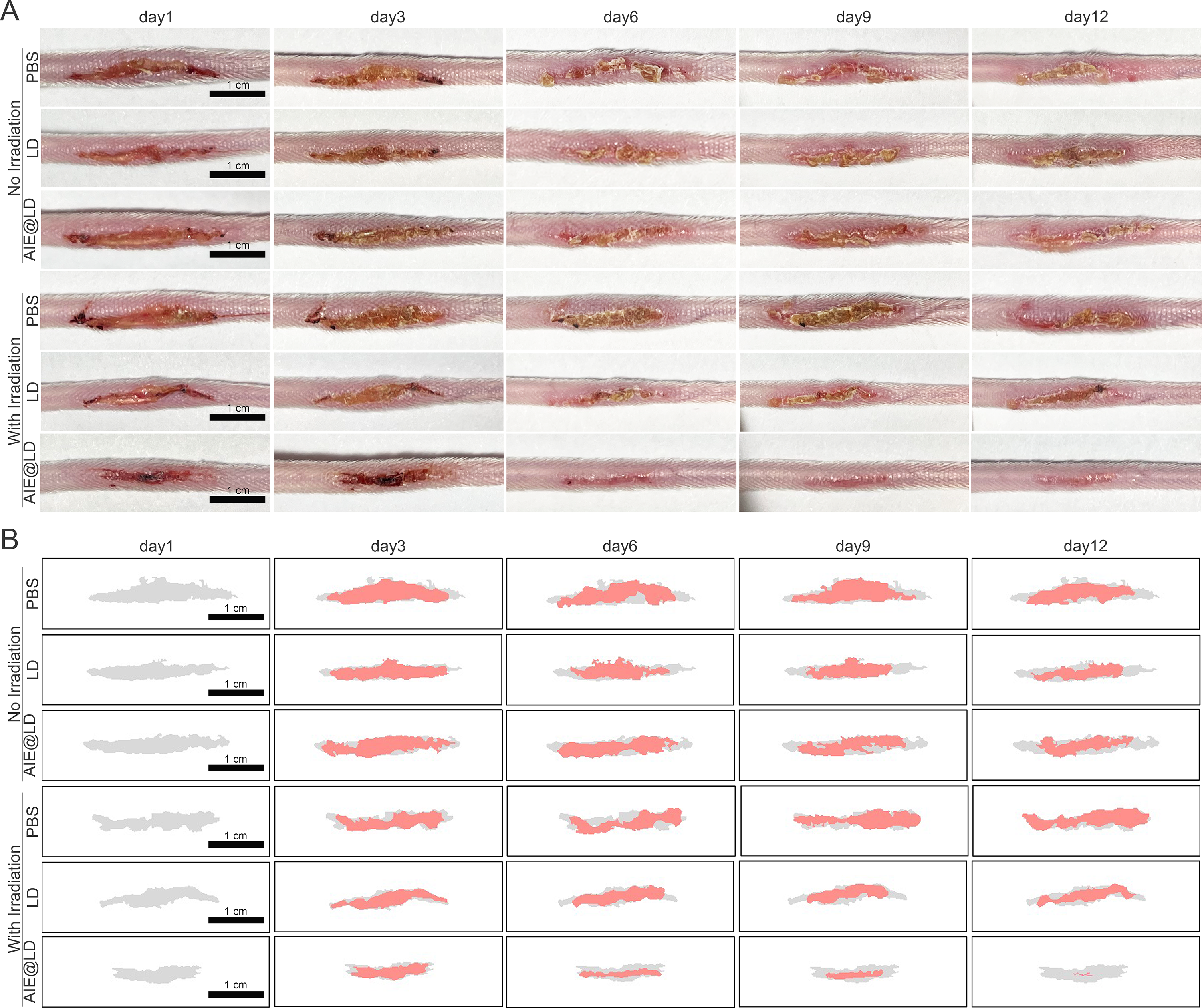


**Figure S12.** (A and B) Photos of wounds and wound trajectory diagrams from different treatment groups (scale = 10 mm). The data are shown as mean ± SDs (n = 3).


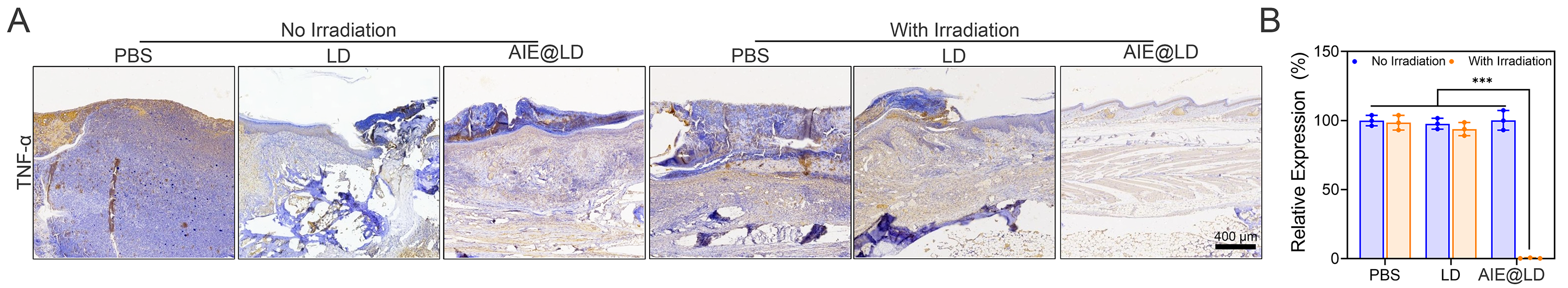


**Figure S13.** Immunohistochemical staining for TNF-a. (A) and statistical analysis (B) of staining intensity in tail lesions subjected to different treatments (scale = 400 µm). The data are shown as mean ± SDs (n = 3). Data analysis was performed using one-way ANOVA. **p* < 0.05, ***p* < 0.01, ****p* < 0.001.


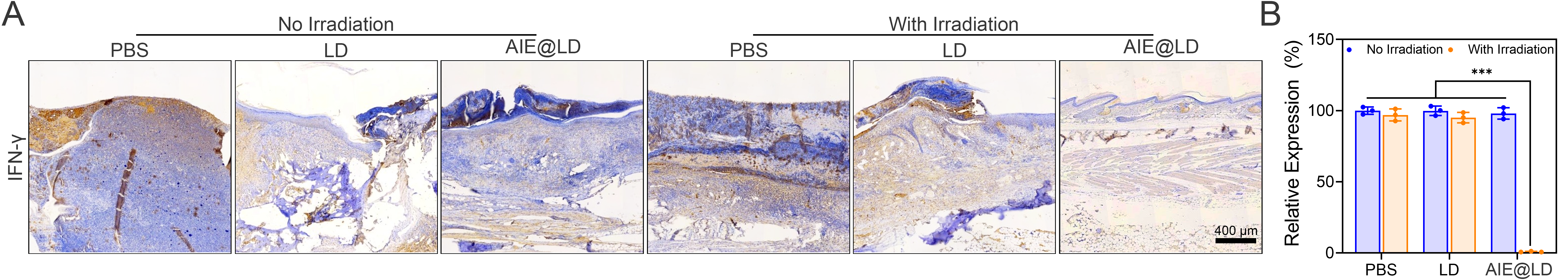
**Figure S14.** **Immunohistochemical staining for IFN-γ.** (A) and statistical analysis (B) of staining intensity in tail lesions subjected to different treatments (scale = 400 µm). The data are shown as mean ± SD**s** (n = 3). Data analysis was performed using one-way ANOVA. **p* < 0.05, ***p* < 0.01, ****p* < 0.001.


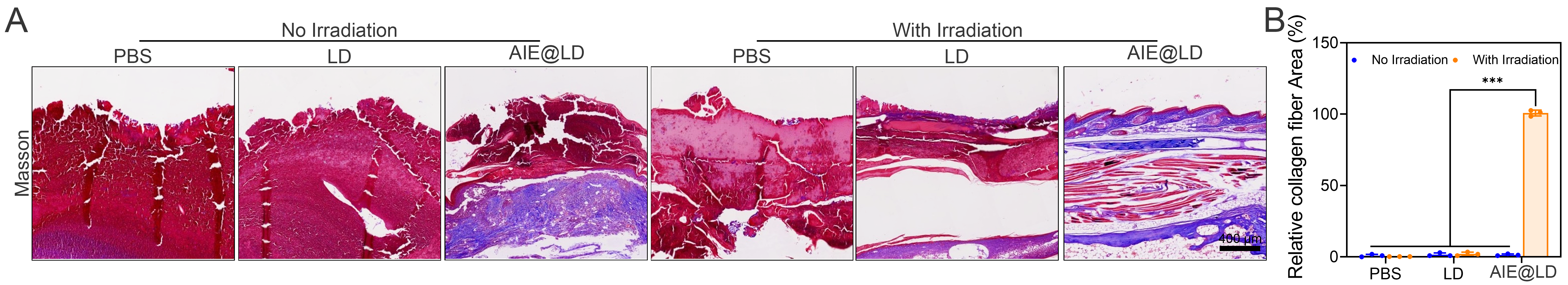


**Figure S15.** Masson staining (A) and statistical analysis (B) of staining intensity in tail lesions subjected to different treatments (scale = 400 µm). The data are shown as mean ± SDs (n = 3). Data analysis was performed using one-way ANOVA. **p* < 0.05, ***p* < 0.01, ****p* < 0.001.


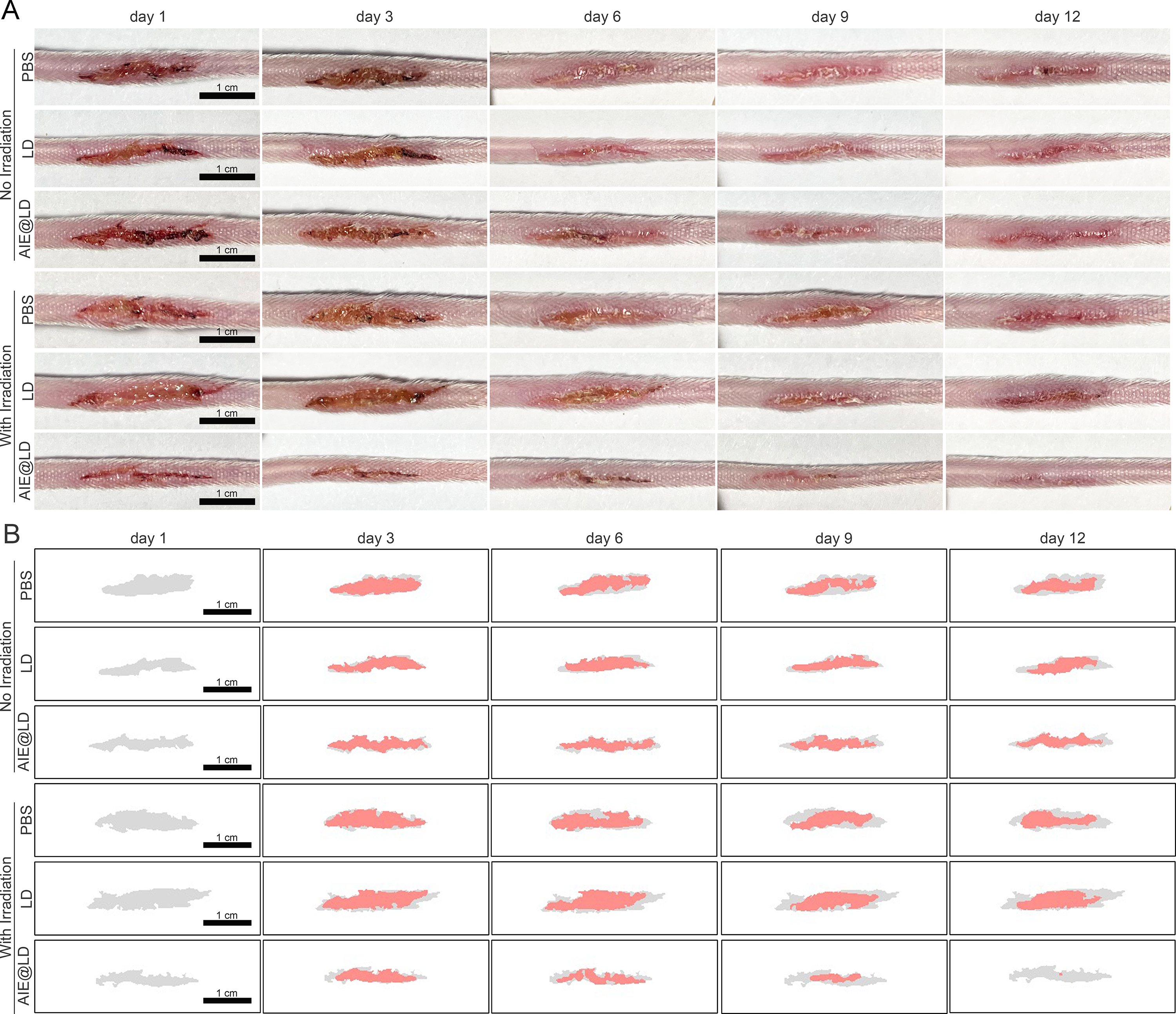


**Figure S16.** (A) After re-inoculating the tails of healthy mice with virus-containing supernatants prepared from the tails of mice with lesions treated differently, take photos of the wound appearance and create (B) a wound trajectory diagram (scale = 10 mm).


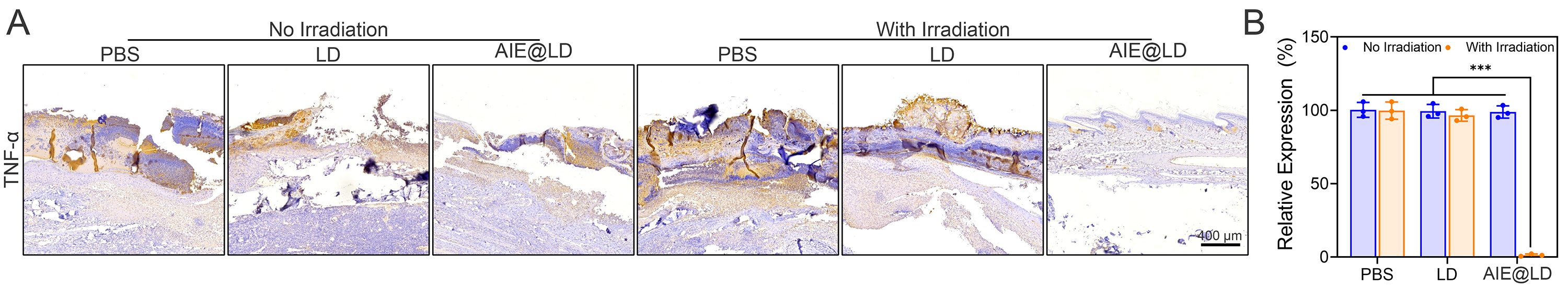


**Figure S17.** **Immunohistochemical staining for TNF-a.** (A) and statistical analysis (B) of staining intensity in blocking virus transmission (scale = 400 µm). The data are shown as mean ± SDs (n = 3). Data analysis was performed using one-way ANOVA. **p* < 0.05, ***p* < 0.01, ****p* < 0.001.


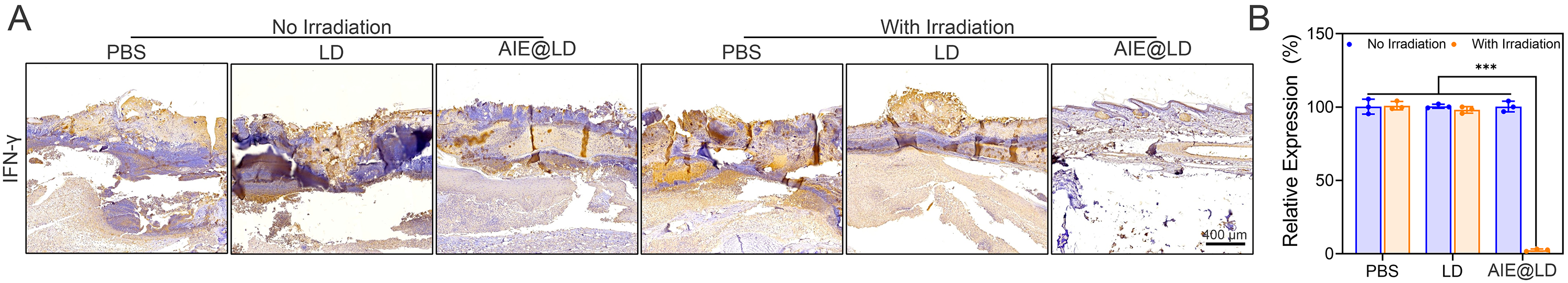


**Figure S18. Immunohistochemical staining for IFN-γ.** (A) and statistical analysis (B) of staining intensity in blocking virus transmission (scale = 400 µm). The data are shown as mean ± SDs (n = 3). Data analysis was performed using one-way ANOVA. **p* < 0.05, ***p* < 0.01, ****p* < 0.001.


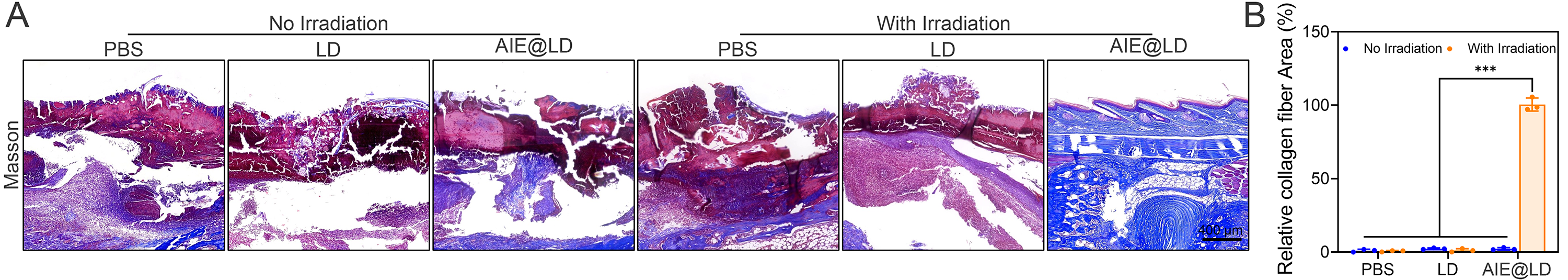


**Figure S19.** Masson staining (A) and statistical analysis (B) of staining intensity in blocking virus transmission (scale = 400 µm). The data are shown as mean ± SDs (n = 3). Data analysis was performed using one-way ANOVA. **p* < 0.05, ***p* < 0.01, ****p* < 0.001.

**
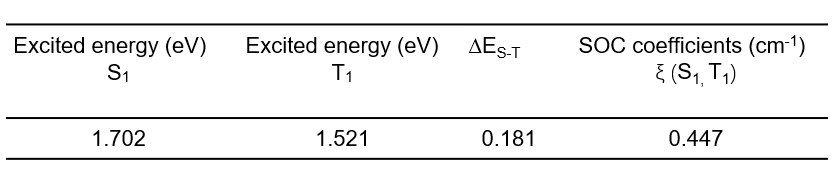
**

**Table S1.** The excited energy and SOC coefficients of TBSMPPy in S_1_ and T_1_.

**Reference**

1 Zhou, Kun, Siyuan Wang, Letian Xu*, et al.* "Aiegen-Based Smart System for Fungal-Infected Wound Monitoring and on-Demand Photodynamic Therapy." *Matter* 6, 10 (2023): 3449-62.
